# Immune and Metabolic Regulation Mechanism of Dangguiliuhuang Decoction against Insulin Resistance and Hepatic Steatosis

**DOI:** 10.3389/fphar.2017.00445

**Published:** 2017-07-07

**Authors:** Hui Cao, Lingling Tuo, Yali Tuo, Ziyun Xia, Rong Fu, Yang Liu, Yihong Quan, Jue Liu, Zhihong Yu, Ming Xiang

**Affiliations:** ^1^Department of Pharmacology, School of Pharmacy, Tongji Medical College, Huazhong University of Science and TechnologyWuhan, China; ^2^Department of Traditional Chinese Medicine, The Central Hospital of Wuhan, Tongji Medical College, Huazhong University of Science and TechnologyWuhan, China; ^3^Department of Pharmacy, China Pharmaceutical UniversityNanjing, China; ^4^Synergy Innovation Center of Biological Peptide Antidiabetics of Hubei Province, School of Life Science, Wuchang University of TechnologyWuhan, China

**Keywords:** Dangguiliuhuang decoction, insulin resistance, hepatic steatosis, dendritic cells, regulatory T cells, metabolism

## Abstract

Dangguiliuhuang decoction (DGLHD) is a traditional Chinese medicine (TCM) formula, which mainly consists of angelica, radix rehmanniae, radix rehmanniae praeparata, scutellaria baicalensis, coptis chinensis, astragalus membranaceus, and golden cypress, and used for the treatment of diabetes and some autoimmune diseases. In this study, we explored the potential mechanism of DGLHD against insulin resistance and fatty liver *in vivo* and *in vitro*. Our data revealed that DGLHD normalized glucose and insulin level, increased the expression of adiponectin, diminished fat accumulation and lipogenesis, and promoted glucose uptake. Metabolomic analysis also demonstrated that DGLHD decreased isoleucine, adenosine, and cholesterol, increased glutamine levels in liver and visceral adipose tissue (VAT) of ob/ob mice. Importantly, DGLHD promoted the shift of pro-inflammatory to anti-inflammatory cytokines, suppressed T lymphocytes proliferation, and enhanced regulatory T cells (Tregs) differentiation. DGLHD also inhibited dendritic cells (DCs) maturation, attenuated DCs-stimulated T cells proliferation and secretion of IL-12p70 cytokine from DCs, and promoted the interaction of DCs with Tregs. Further studies indicated that the changed PI3K/Akt signaling pathway and elevated PPAR-γ expression were not only observed with the ameliorated glucose and lipid metabolism in adipocytes and hepatocytes, but also exhibited in DCs and T cells by DGLHD. Collectively, our results suggest that DGLHD exerts anti-insulin resistant and antisteatotic effects by improving abnormal immune and metabolic homeostasis. And DGLHD may be a novel approach to the treatment of obesity-related insulin resistance and hepatic steatosis.

## Introduction

Obesity is defined as a pathological condition associated with numerous health problems and a reduced life expectancy, reaching epidemic proportions worldwide (Chang et al., 2015). Current trends predict that the levels of obesity and overweight will increase from 33% of the global population in 2005 to 57.8% by 2030 (Pal et al., 2016). The health consequences are significant because obesity can lead to insulin resistance, type 2 diabetes, fatty liver disease, cardiovascular disease, and certain forms of cancer (Hotamisligil, 2006).

It is widely accepted that obesity is associated with low-grade inflammation, which inhibits insulin signaling activity in adipose tissue and liver by interfering with the function of insulin receptor substrate (IRS)-1, peroxisome proliferator activated receptor (PPAR)-γ, and phosphatidyl inositol 3-kinase (PI3K)/protein kinase B (Akt) signaling (Ye, 2013). Impairment of insulin signaling is central to the development of insulin resistance, leading to the development of type 2 diabetes and liver disease. Accumulating evidence has shown that the impaired insulin signaling pathway, together with inflammatory responses, promotes hepatic lipid biosynthesis and steatosis, which in turn contribute to chronic hepatic inflammation and insulin resistance (Wang et al., 2016). Hence, inflammation, insulin resistance, and hepatic steatosis are intertwined pathological events that are commonly presented in obese individuals.

However, inflammation is not the only mechanism of insulin resistance and fatty liver in obesity, various abnormalities of lipid metabolism can also impair insulin signaling. These lipotoxic effects include the production of intracellular lipid mediators, such as diacylglycerols, as well as more direct effects of circulating saturated fatty acids (SFAs) (Glass and Olefsky, 2012). A lipid-rich environment could stimulate IKKβ/NF-κB and JNK1/AP-1 pathways as well as cytokine secretion in immune cells, amplifying proinflammatory effects of SFAs in adipose tissues (Glass and Olefsky, 2012). Therefore, lipid abnormalities and chronic inflammation are closely interconnected and each can amplify the other in the *in vivo* pathophysiologic setting.

Various types of immune cells participate in the development of obesity-related inflammatory and metabolic diseases, including both anti-inflammatory cells (regulatory T cells (Tregs) and eosinophils) and pro-inflammatory cells (macrophages, dendritic cells (DCs), neutrophils, CD8^+^T cells, and B cells) (Lee and Lee, 2014). Tregs exert an important role in the maintenance of immunological self-tolerance (Ilan et al., 2010). Yaron I et al have reported that the number of adipose tissue Tregs is decreased by obesity, and that a boost of these cells in obese mice can improve insulin sensitivity and fat accumulation (Osborn and Olefsky, 2012). Moreover, the expression of PPAR-γ in visceral adipose tissue (VAT) Tregs was essential for restoration of insulin sensitivity in obese mice (Cipolletta et al., 2012).

While, accumulating evidence suggests that the functional status of DCs is involved in the promotion of Tregs differentiation (Hubert et al., 2007). DCs, the most powerful antigen-presenting cells, play an essential role in the development of innate and adaptive immune responses as well as in the regulation of immune homeostasis (Everts and Pearce, 2014). It has been confirmed that depletion of DCs by using CD11c-DTR transgenic mice ameliorates obesity-related inflammation and insulin resistance, and decreases hepatic steatosis and lipid metabolism-related gene expression in the liver (Lee and Lee, 2014). Thus, Tregs and DCs actually exert pivotal roles in the development of obesity-related inflammation, insulin resistance, and hepatic steatosis.

Alterations in cellular metabolism underlie the capacity of immune cells to perform specific functions (Pearce and Pearce, 2013). For example, the induction of glycolysis is important for DCs to acquire immunogenic properties to prolong their survival following activation, while increased mitochondrial metabolism and oxidative phosphorylation are necessary for DCs to express tolerogenic functions (Everts et al., 2014; Everts and Pearce, 2014). Similarly, alterations in glucose and fatty acid metabolism also affect T cell differentiation and function (Fox et al., 2005; van der Windt and Pearce, 2012). Cell metabolism is not only a source of support for cell differentiation and function, but also specific products of metabolic pathways act as signaling molecules to trigger cellular responses. Immune cells are also able to respond directly to metabolites released locally by the surrounding tissue (Pearce and Everts, 2015). These and other studies implicate metabolic reprogramming is an important feature of immune cell activation.

Traditional Chinese medicine (TCM) formulae, often composed of mixtures of biologically active compounds, have a medical practical history for more than 2000 years, and are widely used for a variety of diseases (Tang et al., 2008). Dangguiliuhuang decoction (DGLHD) is a TCM formula first recorded in “Lan Shi Mi Cang” for the treatment of diabetes and hyperthyroidism. The decoction consists of a roughly equal mixture of angelica, radix rehmanniae, radix rehmanniae praeparata, scutellaria baicalensis, coptis chinensis, and golden cypress, with 2 fold higher levels of astragalus membranaceus (Liu et al., 2015). The efficacies of these components in DGLHD are described in Table S1. DGLHD ameliorates the hyperglycemia and hyperlipidmia, and confers β cell protection and insulin sensitization. Modern clinical reports have revealed that DGLHD has protective effects on inflammatory conditions, including diabetes and its complications (Liu et al., 2015). However, the underlying mechanism for these anti-diabetic effects of DGLHD has not been clarified. Therefore, this study was designed to investigate the involvement of altered DCs and Tregs function and of improved metabolic profiles in hepatic and adipose tissues in the anti-diabetic effects of DGLHD in ob/ob mouse model of insulin resistance and hepatic steatosis.

## Materials and methods

### Extraction of DGLHD

Dangguiliuhuang decoction (DGLHD) was provided by the Department of Traditional Chinese Medicine, the Central Hospital of Wuhan. According to the 2015 Edition of the Chinese pharmacopeia, the formula contains angelica sinensis, radix rehmanniae, radix *rehmanniae preparata, radix scutellariae, rhizoma coptidis, cortex phellodendri* and *radix astragali* at the ratio of 1:1:1:1:1:1:2. The compounds are water-soluble, so we soaked the DGLHD extract in distilled water and condensed the solution to a final concentration of 0.13 g/ml. For high-performance liquid chromatography (HPLC) analysis and biological studies, the 0.13 g/ml nominal concentration decoction was filtered (0.2 μm), sterilized, and diluted or concentrated in the proper buffer or medium to the required final concentration.

### Chromatography

Quality control of DGLHD was analyzed by HPLC on a reverse-phase analytical column (Agilent TC-C18, 4.6 × 250 mm, Agilent Technologies). The mobile phase was made up of (A) 100% water with 0.1% phosphoric acid and (B) 100% acetonitrile. Gradient elution from 5 to 45% B for 40 min was used at 10 min intervals. The flow rate was 1 mL/min and the detection wavelength was 280 nm. Chemical standards included calycosin-7-glucoside, verbascoside, ferulic acid, coptisine, berberine, baicalin, wogonoside, baicalein, and wogonin purchased from the National Institutes of Food and Drug Control (Beijing, China). Their purity was verified by HPLC. And GW9662 (PPAR-γ antagonist) was purchased from MedChem Express (Shanghai, China).

### Induction of insulin resistance in 3T3-L1 adipocytes and of steatosis in HepG2 cells

3T3-L1 preadipocytes and HepG2 cells were obtained from Cell Center of Tongji Medical College. They were cultured in DMEM medium supplemented with 10% heat-inactivated fetal bovine serum (FBS, Gibco, USA), 100 U/ml penicillin, and 100 ug/ml streptomycin (Gen-view Scientific Inc., USA) at 37°C and 5% CO_2_.

To yield insulin-resistant 3T3-L1 adipocytes, preadipocytes were induced into mature adipocytes by incubation in DMEM containing 10 μg/mL insulin, 1 μM dexamethasone (DEX), and 0.5 mM isobutylmethylxanthine (IBMX) for 48 h. Subsequently, 3T3-L1 adipocytes were cultured in DMEM with 10 μg/mL insulin and 1.0 μM DEX for 96 h. For establishing a steatosis-like phenotype, HepG2 cells were incubated in DMEM medium with 1 mM oleic acid and 1 mM palmitic acid for 24 h.

### Ethics statement

Animal experiments were approved by the Institutional Animal Care and Use Committee of Tongji Medical College, Huazhong University of Science and Technology. Male Lep^ob^/Lep^ob^ (ob/ob) mice and male C57BL/6J mice were purchased from Beijing Huafukang Bio-Technology Co., Ltd., and maintained at the experimental animal center of Tongji Medical College (Huazhong University of Science and Technology, China) under specific pathogen-free conditions. The animals were housed at 23 ± 2°C in a 12 h light/dark cycle with free access to standard diet and water.

### Animal study

Obesity-related insulin resistance and hepatic steatosis were evaluated in spontaneously obese ob/ob mice. Either a solution of DGLHD (1.5, 3.0, 6.0 g/kg) or an equivalent volume of water were administered intragastrically to ob/ob mice once a day for 8 weeks. A Tregs neutralizing mouse model was established by injecting intraperitoneally LE/AF-purified anti-mouse CD25 antibody (Tianjin Sungene Biotech Co., Ltd., Tianjin, China) into ob/ob mice, at a dose of 125 μg/mouse once a week for 3 weeks. To investigate adverse effects of DGLHD, C57BL/6J mice were treated with water or DGLHD (6.0 g/kg) by intragastrical gavage for 8 weeks.

Body weights and mean food consumption of ob/ob mice in each group were monitored once a week for 8 weeks. After the prescribed time period, mice were fasted overnight and then operated appropriately for tissues and serum.

### Glucose tolerance test and insulin tolerance test

Oral glucose tolerance tests (OGTT) and insulin tolerance tests(ITT)were performed after ob/ob mice received DGLHD or water for 8 weeks. For the OGTT, mice were fasted for 16 h and given D-glucose (2 g/kg) intragastrically. Blood glucose was then measured at 0, 15, 30, 60, 90, and 120 min after glucose administration using a Contour blood glucose meter (Bayer, Pittsburgh, PA). For ITT, mice were fasted for 6 h, then injected intraperitoneally with insulin (1 U/kg). Blood glucose was then measured at 0, 15, 30, 60, 90, and 120 min after insulin injection.

### Histology and morphological evaluation

Liver and VAT were fixed in 10% formalin, embedded in paraffin, and stained with hematoxylin and eosin (H&E). Hepatic lipid content was also determined by Oil Red O staining. Steatosis areas were quantified by histomorphometry using a computerized image analysis system. Steatosis score was assigned under double-blinded conditions and the degree of steatosis was determined. Scores of 0, 1, 2, and 3 corresponded to steatosis areas of <5, 5–33, 33–66, and >66%, respectively (Kleiner et al., 2005). At least 10 independent fields per sample were evaluated in each treatment group. Insulin expression levels in pancreatic islets were detected by immunohistochemistry.

### Biochemical analysis and enzyme-linked immunosorbent assay (ELISA)

The concentrations of free fatty acid (FFA), triglyceride (TG), and total cholesterol (TC) in serum, liver and HepG2 cells were measured by commercial detection kits (Nanjing Jiancheng Bioengineering Institute, Nangjing, China) according to the manufacturer's protocols. Glucose levels in the cell suspension were determined using the glucose oxidase method.

Serum from DGLHD-treated ob/ob mice and control mice, and supernatant from T cells, DCs, and 3T3-L1 and HepG2 cell lines—with or without DGLHD treatment—were reserved at −80°C until quantification. The concentrations of interleukin (IL)-10, transforming growth factor (TGF)-β1, interferon (IFN)-γ, tumor necrosis factor (TNF)-α, IL-12p70, insulin, and adiponectin were measured by commercial ELISA kits (Anhui Qiaoyi Biological Technology Co., Ltd., Hefei, China).

### DCs generation and mixed leukocyte reaction (MLR)

Dendritic cells (DCs) derived from bone marrow were prepared as reported previously (Wang J. et al., 2015). Briefly, bone marrow cells were cultured in RPMI-1640 medium containing 10% FBS, 100 U/ml penicillin-100 ug/ml streptomycin, 20 ng/ml mouse granulocyte-monocyte colony-stimulating factor (mGM-CSF, Signalway Antibody LLC, USA), and 10 ng/mL mouse IL-4 (Signalway Antibody LLC, USA). Fresh culture medium was added on day 3, and the medium was refreshed on day 5. At day 7, semi-adherent cells were collected as DCs.

The Mixed lymphocyte reaction (MLR) was used to assess the immunostimulatory function of DCs and was estimated using a 3-(4, 5-dimethyl-2-thiazolyl)-2, 5-diphenyl-2-H-tetrazolium bromide (MTT) assay. In brief, DCs from ob/ob mice as stimulators were treated with 0.5 mg/mL mitomycin C to prevent proliferation. Then DCs and allogeneic T cells were co-cultured at the ratio of 1:10 for 48 h.

### Generation and proliferation of T cells

T cells were isolated from spleen by anti-CD4 immunomagnetic beads (Miltenyi Biotec Inc. Auburn, CA, USA) and cultured in RPMI 1640 supplemented with 10% FBS. T cells were stimulated with anti-CD3 antibody (5 μg/ml) and anti-CD28 antibody (5 μg/ml) in the absence or presence of DGLHD for 3 days. T lymphocyte proliferation was evaluated by MTT assay at 490 nm.

### Flow cytometry analysis

Cell suspensions were prepared from spleen, lymph nodes, VAT, or liver of ob/ob mice as previously described (Chang et al., 2015; Xiang et al., 2016). DC single-cell suspensions were obtained from bone marrow following standard procedures. Cells were stained with antibodies against the following cell surface antigens: fluorescein isothiocyanate (FITC)-conjugated anti-CD86, allophycocyanin (APC)-conjugated anti-CD11c, phycoerythrin (PE)-conjugated anti-MHC-II and PE-conjugated anti-PD-L1 (eBioscience, USA); APC-conjugated anti-Foxp3, FITC-conjugated anti-CD4, PE-conjugated anti-CD25 (eBioscience, USA); APC-conjugated anti-CD3, PE-conjugated anti-PD-1, and PE-conjugated anti-TCRβ (eBioscience, USA). Samples were detected on a BD LSRII flow cytometer or BD C6 (BD Pharmingen), and data were analyzed with Flowjo software, version 10.1.

### Western blot analysis

Cells or tissues were lysed in Radio-Immunoprecipitation Assay (RIPA) buffer, and protein concentration was quantified with a BCA Protein Quantitation Kit (Thermo Fisher Scientific Inc., USA). Lysate samples were separated by SDS-polyacrylamide gels (SDS-PAGE) and transferred to polyvinylidene difluoride (PVDF) membranes, blocked with 5% non-fat milk, and incubated with indicated antibodies overnight at 4°C. Appropriate horseradish peroxidase (HRP)-conjugated secondary antibodies were applied for 1–2 h at room temperature, prior to detection with an enhanced chemiluminescence kit. Polyclonal antibodies to anti-PI3K, anti-p-PI3K (Ser 192), anti-Akt, anti-p-Akt (Thr 183/Tyr 185), and anti-PPAR-γ were purchased from Cell Signaling Technology (CST, Danfoss, Massachusetts, USA). The amount of protein expression was normalized by β-actin.

### RNA extraction and quantitative real-time PCR

Quantitative real-time PCR (qRT-PCR) was performed as described previously (Xiang et al., 2016). In brief, total RNA was isolated from cells, liver, or VAT using TRizol reagent, and reverse transcribed with the Revert Aid First strand cDNA synthesis kit (Invitrogen, USA) with random primers based on the manufacturer's instructions. qRT-PCR was conducted using a SYBR Green Master Mix (Bioer, Hangzhou Bioer technology co., Ltd.,) on CFX Connect Real-Time PCR System (Bioer, Hangzhou Bioer technology Co., Ltd.,). Gene expressions were normalized to those of β-actin. Primer sequences are listed in Table S2.

### Metabolite profiling

For widely-targeted metabolomic analyses—of liver and VAT obtained from control and DGLHD (6.0 g/kg)-administrated mice—a liquid chromatography-electrospray ionization-tandem mass spectrometry (LC-ESI-MS/MS) system was applied. The samples were pre-processed before detection according to previous report (Chen et al., 2016). Simply, hepatic and visceral adipose tissues were freeze-dried, crushed, extracted, and reconstituted in appropriate solvents. Quantification of metabolites was performed using a scheduled multiple reaction monitoring method. The relative signal intensities of metabolites were normalized by first dividing them by the intensities of the internal standard and then log2 transforming them for further normalization. We considered metabolites with ≥1.5-fold changes or ≤ 0.5-fold changes as differential expressions. Related metabolic pathway-enhancement analyses were investigated using MBRole and KEGG database (Chagoyen and Pazos, 2011).

### Statistical analysis

Data are presented as mean ± SEM. Statistical analysis was performed in GraphPad Prism 5. Statistical significance involved in more than two groups were analyzed by the one-way ANOVA. Statistical analysis between two groups were assessed by Student's *t*-test. *P* < 0.05 were considered statistically significant.

## Results

### Chemical analysis of DGLHD

High-performance liquid chromatography (HPLC) analysis identified four major compounds in the DGLHD decoction: 75.17 mg/L berberine, 160.70 mg/L baicalin, 86.53 mg/L wogonoside, and 49.45 mg/L wogonin (Figure S1).

### DGLHD reduces hepatic steatosis in ob/ob mice

**Figure 1:**
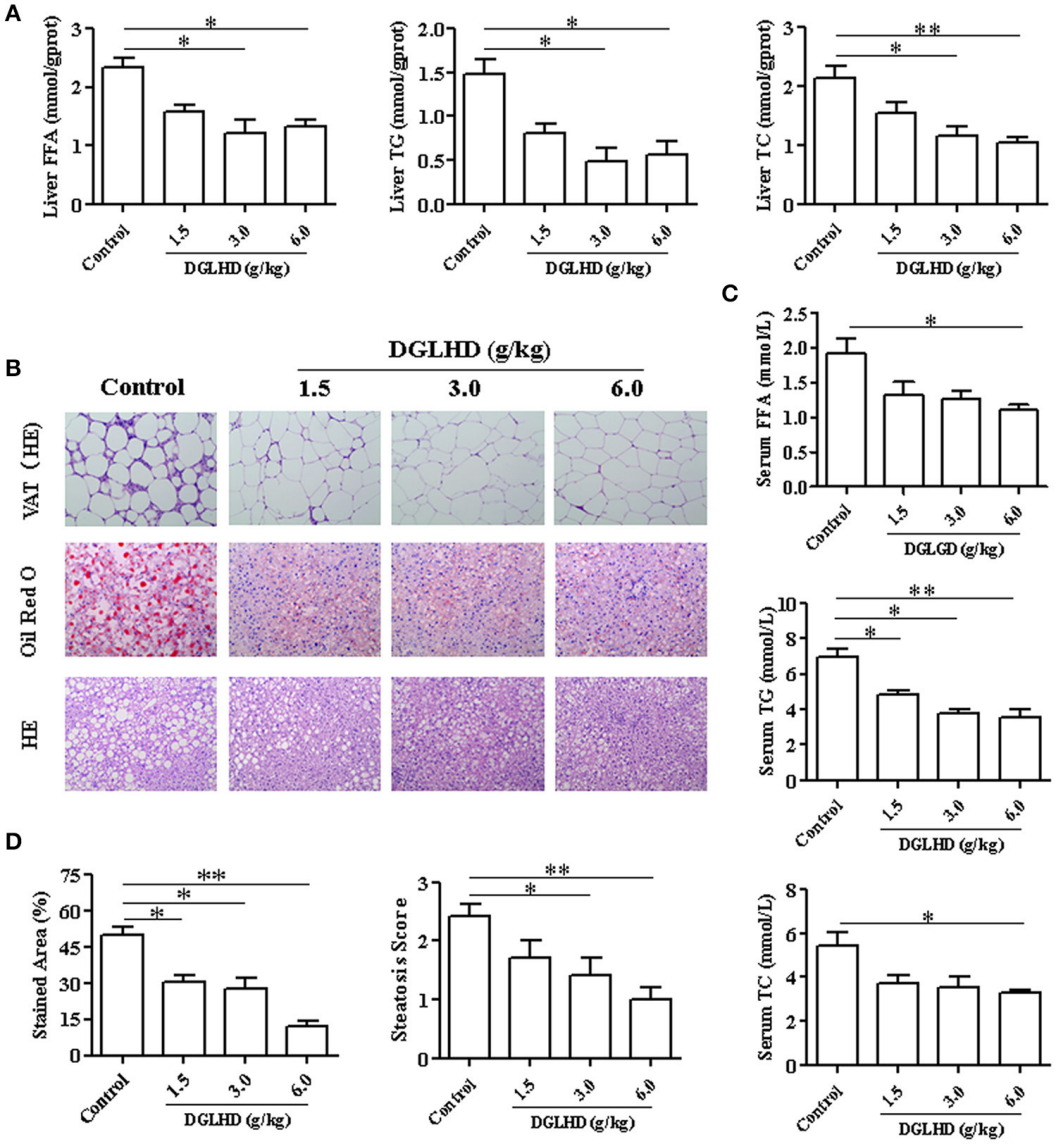
Effects of DGLHD on fat accumulation in ob/ob mice. Ob/ob mice were treated daily with water or DGLHD at 1.5, 3.0, or 6.0 g/kg by intragastric gavage for 8 weeks (*n* = 8 for each group). **(A)** The levels of free fatty acid (FFA), triglyceride (TG), and total cholesterol (TC) in liver. **(B)** Representative photomicrographs of VAT stained with hematoxylin-eosin; Representative images of liver sections stained with Oil Red O or hematoxylin-eosin (×200). **(C)** Concentrations of FFA, TG, and TC in serum. **(D)** Steatosis area and scores of liver in different groups. Data are expressed as mean ± SEM (*n* = 5). ^*^P < 0.05, ^**^P < 0.01 vs. Control.

The ob/ob mouse carries a spontaneous mutation in the leptin gene, which results in a leptin deficiency, subsequently leading to hyperphagia, inactivity, obesity, insulin resistance, and hepatic steatosis (Kanuri and Bergheim, 2013). We thus adopted ob/ob mice as an animal model to investigate effects of DGLHD in regulating lipid homeostasis. Results showed that treatment with DGLHD ameliorated hepatic steatosis, decreased FFA, TG, and TC levels in liver and serum, and reduced the weight of liver and VAT (Figure 1 and Figures S2C,D). However, DGLHD administration did not exert notable influences on body weight or food intake (Figures S2A,B), though it did decrease adipocyte size (Figure 1B). DGLHD also significantly attenuated the crown-like structures in VAT without affecting normal morphology (Figure 1B). These results provided preliminary evidence that DGLHD reduced fat accumulation and improved lipid metabolism in ob/ob mice.

### DGLHD improves insulin resistance in ob/ob mice

**Figure 2:**
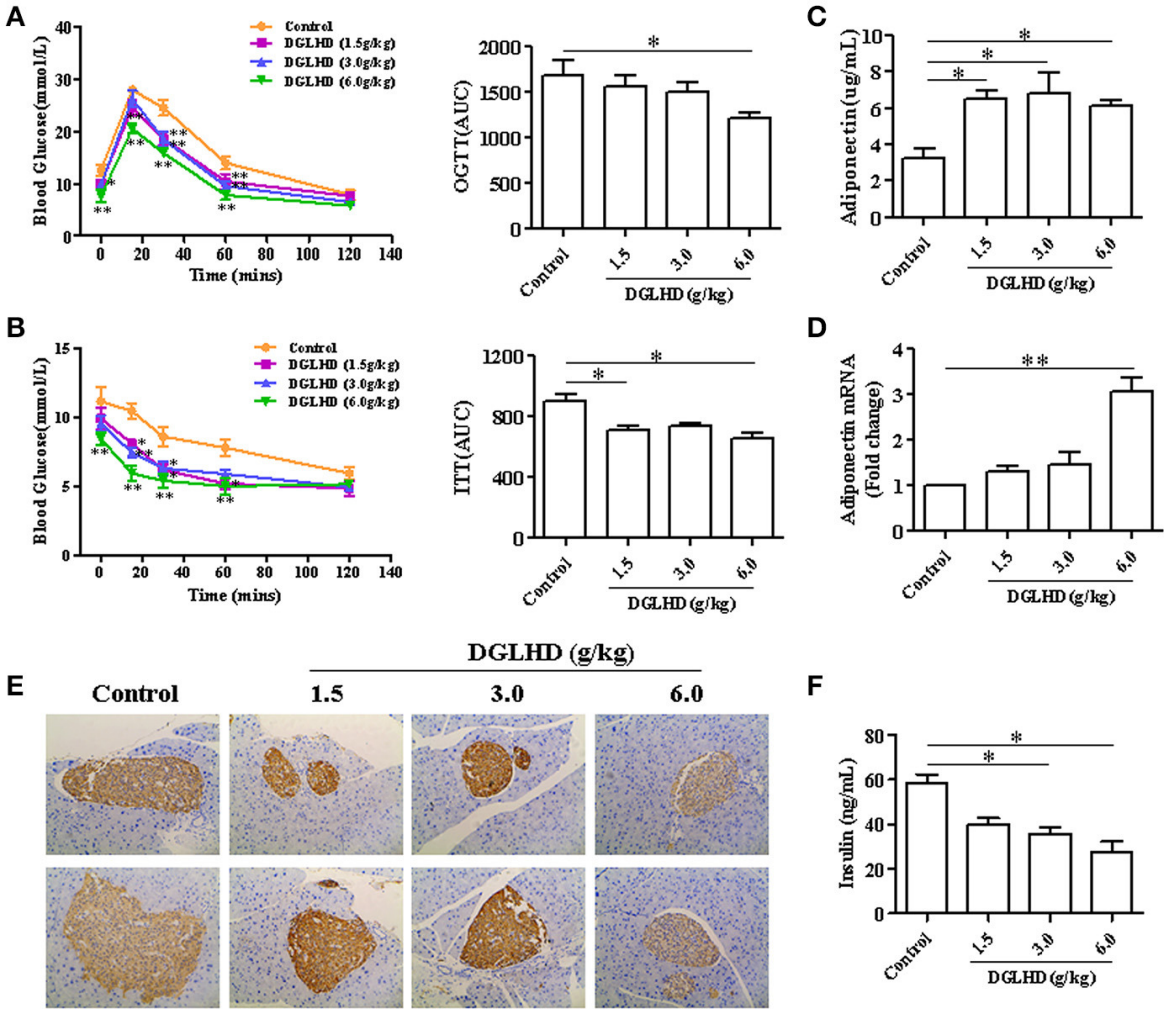
DGLHD improves glucose tolerance and insulin sensitivity in ob/ob mice. Oral glucose tolerance tests (OGTTs) **(A)** or insulin tolerance tests (ITTs) **(B)** were performed in mice after 16 or 6 h of food deprivation, respectively. Blood glucose levels were measured before (time 0) and after (15, 30, 60, and 120 min) glucose or insulin administration in ob/ob mice treated daily with water or DGLHD for 8 weeks. AUC, area under the curve of OGTT or ITT. **(C,D)** Expression levels of adiponectin in serum and VAT. **(E)** Representative images of insulin immunohistochemical staining in pancreatic islets (×200). **(F)** Insulin concentrations in serum were assayed by ELISA. Data are presented as mean ± SEM (*n* = 5). ^*^P < 0.05, ^**^P < 0.01 vs. Control.

To investigate possible insulin-sensitizing effects of DGLHD, we performed OGTT and ITT on ob/ob mice. As shown in Figures 2A,B, DGLHD improved glucose tolerance and insulin sensitivity, quantified by diminished AUCs for blood glucose during the OGTTs and ITTs. Considering that insulin resistance is associated with insulin hypersecretion by β-cells (Lee and Lee, 2014), we next examined serum insulin levels and histopathological changes of pancreatic islets. The results demonstrated that DGLHD at dose of 6 g/kg markedly lowered serum insulin level and islet size, and decreased insulin secretion of pancreas islets in ob/ob mice (Figures 2E,F), indicating that DGLHD treatment confers islet cell protection. These results are consistent with the view that DGLHD treatment of ob/ob mice normalizes glucose and insulin metabolic control.

Adiponectin is an adipokine with hypoglycemic, antidiabetic, antilipogenic, and anti-inflammatory properties (Gonzalez-Periz et al., 2009). We found that DGLHD treatment markedly increased adiponectin level in serum as well as adiponectin mRNA level in VAT (Figures 2C,D), consistent with positive effects of DGLHD on insulin sensitization. The data thus indicated that improved insulin sensitivity and islet function produced by DGLHD were associated with elevated adiponectin level and secretion.

### DGLHD alters metabolites in liver and visceral adipose tissues

**Figure 3:**
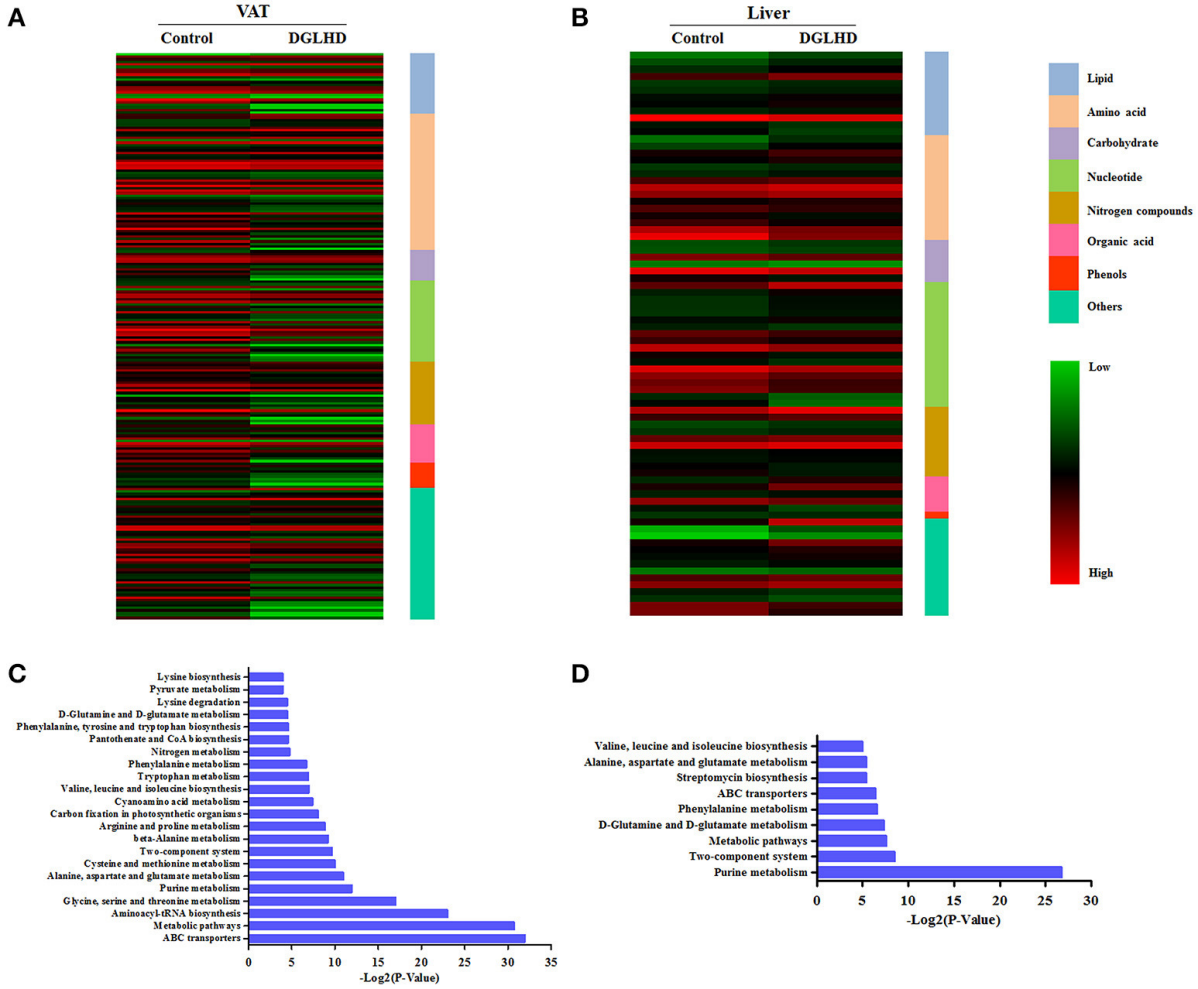
Metabolites in visceral adipose and liver tissues from DGLHD-treated and control mice. The heat map represents the log2 value of the amount of different metabolites with ≥1.5-fold change or ≤0.5-fold change in VAT **(A)** and liver **(B)**. The complete metabolomic profiles are provided in Data Sheet 1. KEGG pathway enrichment analysis for metabolites in VAT **(C)** and liver **(D)** from DGLHD-treated and control mice. The complete data of pathway enrichment analysis are provided in Data Sheet 2.

A metabolomic approach was used to analyze the effect of DGLHD on specific metabolites in ob/ob mice. We chose insulin-responsive and metabolically important organs—liver and adipose tissue—to obtain metabolite profiles. An overview of hepatic and adipose metabolites concentration in DGLHD-treated ob/ob mice and control mice is shown in Table S3. Of the 355 hepatic metabolites detected, 83 metabolites exhibited a significant change (increase or decrease) of >50% in response to DGLHD. Metabolites that showed prominent decreases included isoleucine, adenosine and its derivatives, and cholesterol, while glutamine, phosphocholine, and polyunsaturated fatty acids increased (Figure 3B and Data Sheet 1).

In VAT, DGLHD exposure resulted in prominent declines in metabolites of lipid, amino acids, and nucleotides, including polyunsaturated fatty acids, branched-chain fatty acids (BCAA), aromatic amino acids, and purines, as well as cholesterol and glucose (Figure 3A and Data Sheet 1). Other changes included increases in glycine and glutamine, and decreases in lysophosphatidylcholine, glutamine, and glycine (Figure 3A and Data Sheet 1).

In order to characterize the metabolic alterations effected by DGLHD in more detail, we further conducted pathway-enhancement analysis for different metabolites. We found that treatment of ob/ob mice with DGLHD strongly influenced pathways in adipose tissue that control levels of ABC transporters, metabolism of glycine, serine, threonine, arginine, alanine, glutamate, phenylalanine, and purines, and the biosynthesis of aminoacyl-tRNA (Figure 3C and Data Sheet 2). In liver, DGLHD treatment altered pathways associated with the metabolism of purines, glutamate, glutamine, alanine, and phenylalanine (Figure 3D and Data Sheet 2). Collectively, these results were consistent with the hypothesis that DGLHD ameliorates insulin resistance and hepatic steatosis in ob/ob mice.

### DGLHD alleviates inflammation in ob/ob mice

**Figure 4:**
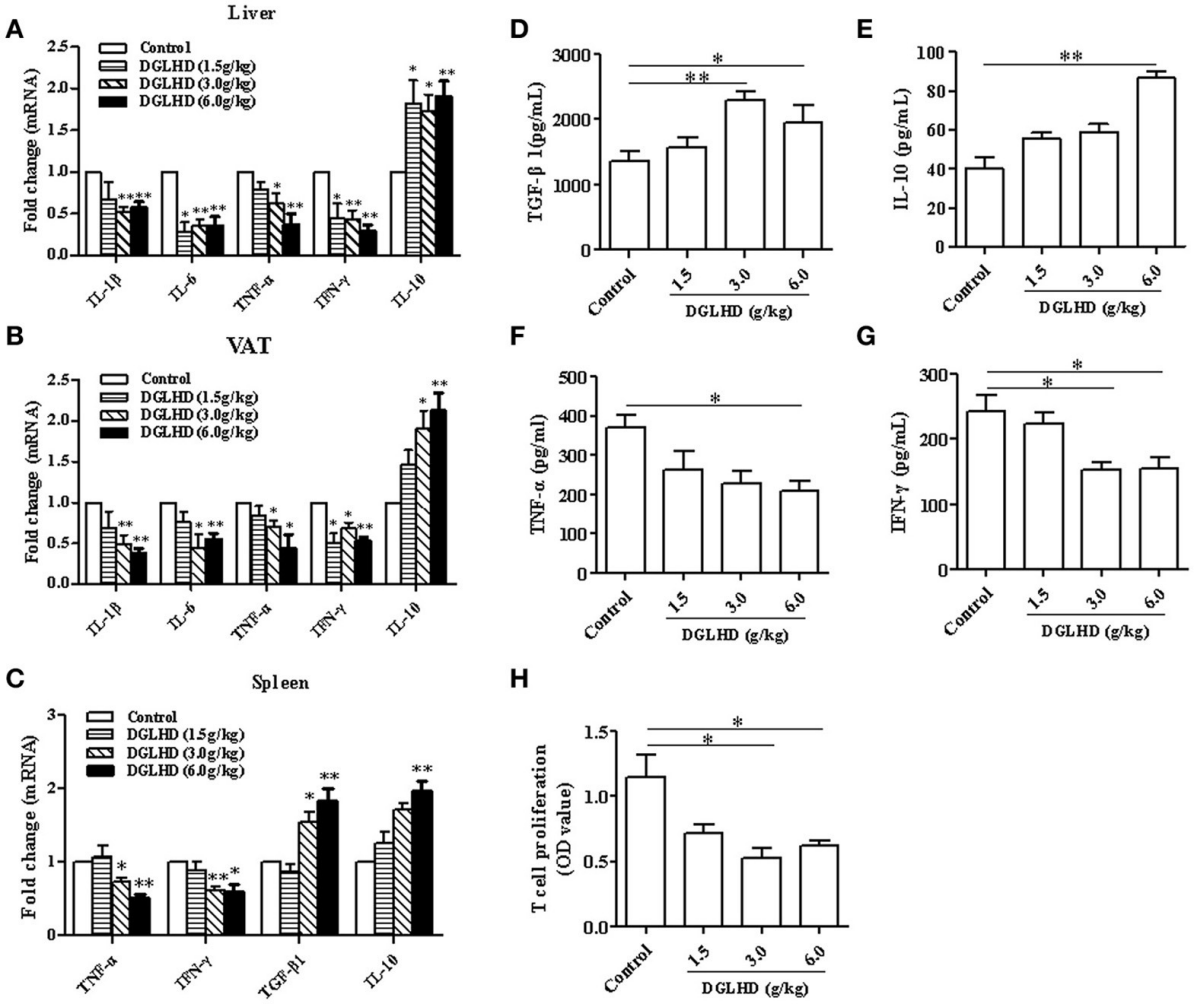
DGLHD diminishes local and systemic inflammation in ob/ob mice. Hepatic and visceral adipose tissues, spleen, and serum were derived from ob/ob mice given water or DGLHD. **(A,B)** Gene expression levels of IL-1β, IL-6, TNF-α, IFN-γ, and IL-10 were determined in liver and VAT by qRT-PCR analyses. **(C)** The mRNA levels of TGF-β1, IL-10, IFN-γ, and TNF-α in splenic T cells. **(D–G)** The productions of TGF-β1, IL-10, IFN-γ, and TNF-α in serum were detected by ELISA. **(H)** Splenic T cell proliferation was measured by MTT assay. β-actin was detected as the internal reference. Data shown are mean ± SEM (*n* = 4–5). ^*^P < 0.05, ^**^P < 0.01 vs. Control.

Given that obesity is considered as a kind of chronic low-grade inflammation characterized by increased cytokine levels in the circulation and at local sites (Lee and Lee, 2014), we examined mRNA levels of the pro-inflammatory cytokines-TNF-α, IFN-γ, IL-1β, IL-6-, and the anti-inflammatory cytokine IL-10 in adipose and hepatic tissues of untreated and DGLHD-treated ob/ob mice. As depicted in Figures 4A,B, mRNA expressions of these pro-inflammatory cytokines were reduced by DGLHD, whereas IL-10 expression was increased. In line with these results, treatment with DGLHD also decreased the secretion of TNF-α and IFN-γ cytokines, and elevated the production of IL-10 and TGF-β1 cytokines in serum (Figures 4D–G). Moreover, DGLHD treatment also decreased TNF-α and IFN-γ mRNA levels, and increased IL-10 and TGF-β1 levels in splenic T cells, while significantly suppressed splenic T cells proliferation (Figures 4C,H).

### DGLHD elevates tregs generation *In vivo* and *In vitro*

**Figure 5:**
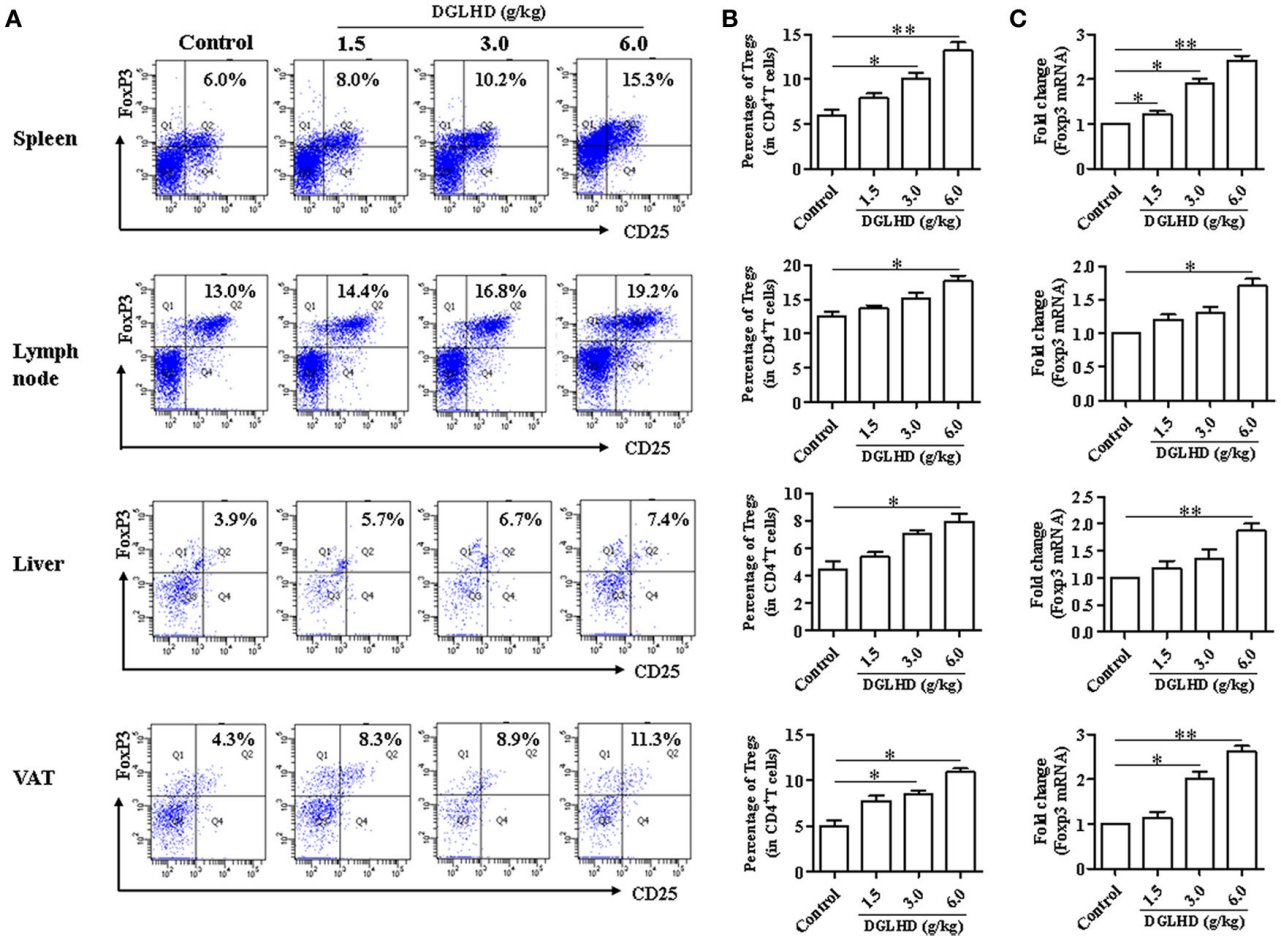
DGLHD promotes the generation of CD4^+^ CD25^+^ Foxp3^+^ Tregs. Spleen, lymph node, liver, and visceral adipose tissues were obtained from ob/ob mice in different groups. **(A,B)** Representative FACS staining for CD25 and Foxp3 on gated CD4^+^ T cells and the percentage of CD4^+^ CD25^+^ Foxp3^+^ Tregs in spleen, lymph node, liver, and visceral adipose tissues. **(C)** RNA was isolated from these tissues and used for qRT-PCR analyses of Foxp3 gene. Data are mean ± SEM (*n* = 4–5). ^*^P < 0.05, ^**^P < 0.01 vs. Control.

**Figure 6:**
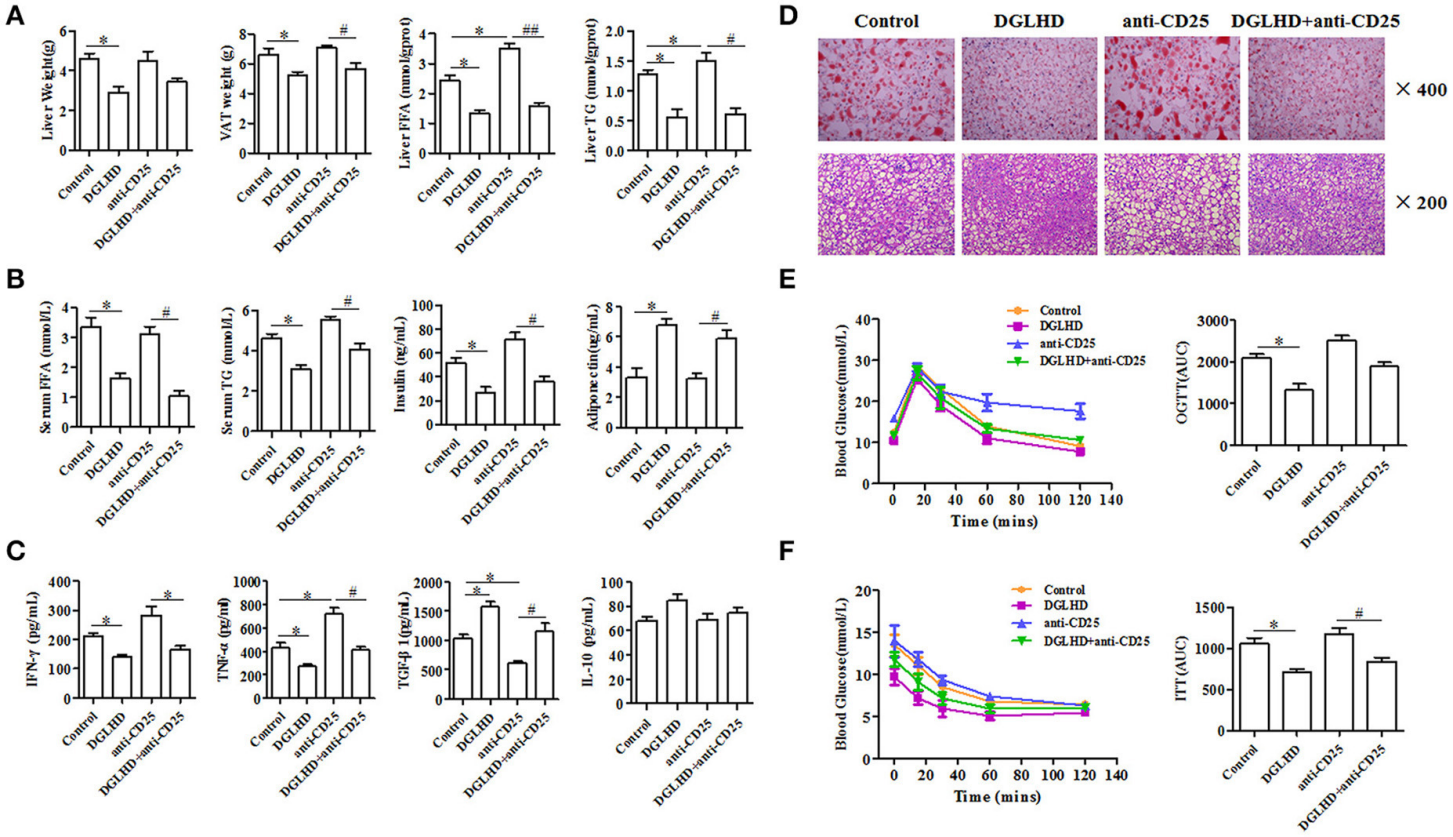
DGLHD reverses the aggravation of insulin resistance and hepatic steatosis in ob/ob mice caused by Tregs depletion. Tregs in ob/ob mice were neutralized with anti-CD25 antibody. **(A)** Liver weight, VAT weight, and the levels of FFA and TG in liver. **(B)** The levels of FFA, TG, insulin and adiponectin in serum. **(C)** The levels of TGF-β1, IL-10, IFN-γ and TNF-α in serum were determined by ELISA. **(D)** The lipid content of liver were detected by Oil Red O and HE staining. **(E)** Oral glucose tolerance tests (OGTTs) or **(F)** insulin tolerance tests (ITTs) were performed in ob/ob mice, respectively. AUC, area under the curve of OGTT or ITT. Data are expressed as mean ± SEM (*n* = 4–5). ^*^P < 0.05 vs. Control; ^#^*P* < 0.05, ^*##*^*P* < 0.01 vs. anti-CD25 group.

To investigate immune cells involved in the anti-inflammatory and immunoregulatory functions of DGLHD on ob/ob mice, we quantified their proportions in spleen, lymph nodes, liver, and VAT by FCM. Administration of DGLHD elevated the percentage of Tregs as well as their Foxp3 gene expression, but displayed little effect on other T cell subtypes (Figure 5 and Figure S3). We also found that DGLHD slightly reduced TCR levels, and increased negative regulatory molecule programmed death 1 (PD-1) in T cells from spleen, lymph node, and liver (Figure S4). Neutralization of Tregs in ob/ob mice strongly reduced the numbers of Tregs in liver, spleen and lymph nodes (Figure S5A). Notably, these reductions were accompanied by aggravated fatty liver, insulin resistance, and inflammation in ob/ob mice (Figure 6), which was consistent with previous reports (Osborn and Olefsky, 2012). Intriguingly, DGLHD treatment reversed these alterations after blocking Tregs (Figure 6 and Figure S5A). To further elucidate the impact of DGLHD on Tregs *in vitro*, we isolated CD4^+^T cells from ob/ob mice. Our *in vitro* results showed that DGLHD inhibited T cells proliferation, contributed to the differentiation of T cells into Tregs, and elicit Th1 to Th2 cytokines shift (Figures S6A–D). Overall, these results showed that Tregs seemed to be critical for ameliorative anti-inflammatory actions of DGLHD in ob/ob mice, and that Tregs may be one of the target cells for DGLHD.

### DGLHD changes DCs characteristics and function *In vivo* and *In vitro*

**Figure 7:**
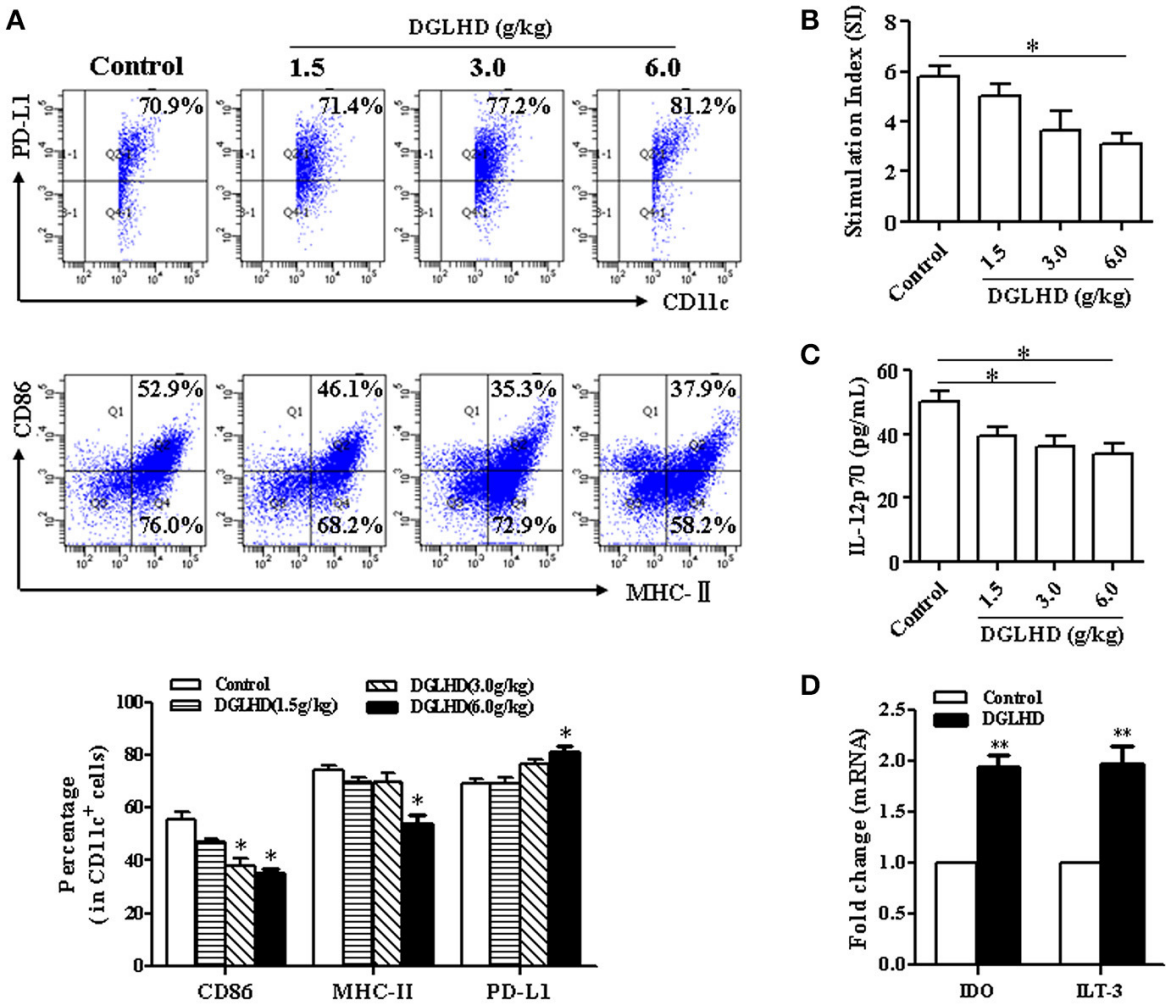
DGLHD modulates DC maturation and function *in vivo*. Bone marrow dendritic cells were obtained from ob/ob mice fed with water or DGLHD. **(A)** The levels of MHC-II, CD86, and PD-L1 in DCs were detected by FCM. **(B)** The stimulatory capacity of T cell proliferation was surveyed by MLR. DCs from DGLHD-treated mice disrupted T cells proliferation. **(C)** Expression of IL-12p70 cytokine in DCs suspension was quantified by ELISA. **(D)** Gene levels of IDO and ILT3 were analyzed by qRT-PCR. Data are representative of three or four independent experiments. Data are expressed as mean ± SEM. ^*^P < 0.05, ^**^P < 0.01 vs. Control.

It has been previously reported that DGLHD can regulate the maturation and function of DCs from NOD mice (Liu et al., 2015). We thus explored whether DCs participate in the anti-inflammatory and anti-insulin resistant effects of DGLHD on ob/ob mice. We found that DGLHD did decrease the level of MHC-II and CD86, and enhanced the expression of negative regulatory molecule programmed death ligand 1 (PD-L1) (Figure 7A). In addition, treatment with DGLHD inhibited DC's stimulation of T cell proliferation, suggesting that DGLHD disrupted the stimulatory capacity of DCs (Figure 7B). Consistently, DGLHD administration also upregulated mRNA levels of indoleamine-2, 3-dioxygenase (IDO), an anti-inflammatory factor expressed by tolerogenic DCs, and immunoglobulin-like transcript (ILT)-3, an inhibitory receptor of DCs (Figure 7D), but significantly antagonized DC-induced production of the cytokine IL-12p70 (Figure 7C). Additionally, DGLHD also inhibited the maturation of DCs in Tregs-neutralized mice (Figure S5B). Consistent with these findings, we also noted that DGLHD inhibited phenotypic maturation, antigen presentation, IL-12p70 secretion *in vitro* (Figures S7A–D). Taken together, these results suggested that DGLHD promoted immune homeostasis and suppressed inflammation in ob/ob mice at least in part by retarding DC maturation and inhibiting their capacity to stimulate T cells.

### DGLHD affects the metabolic factors of DCs and T cells

**Figure 8:**
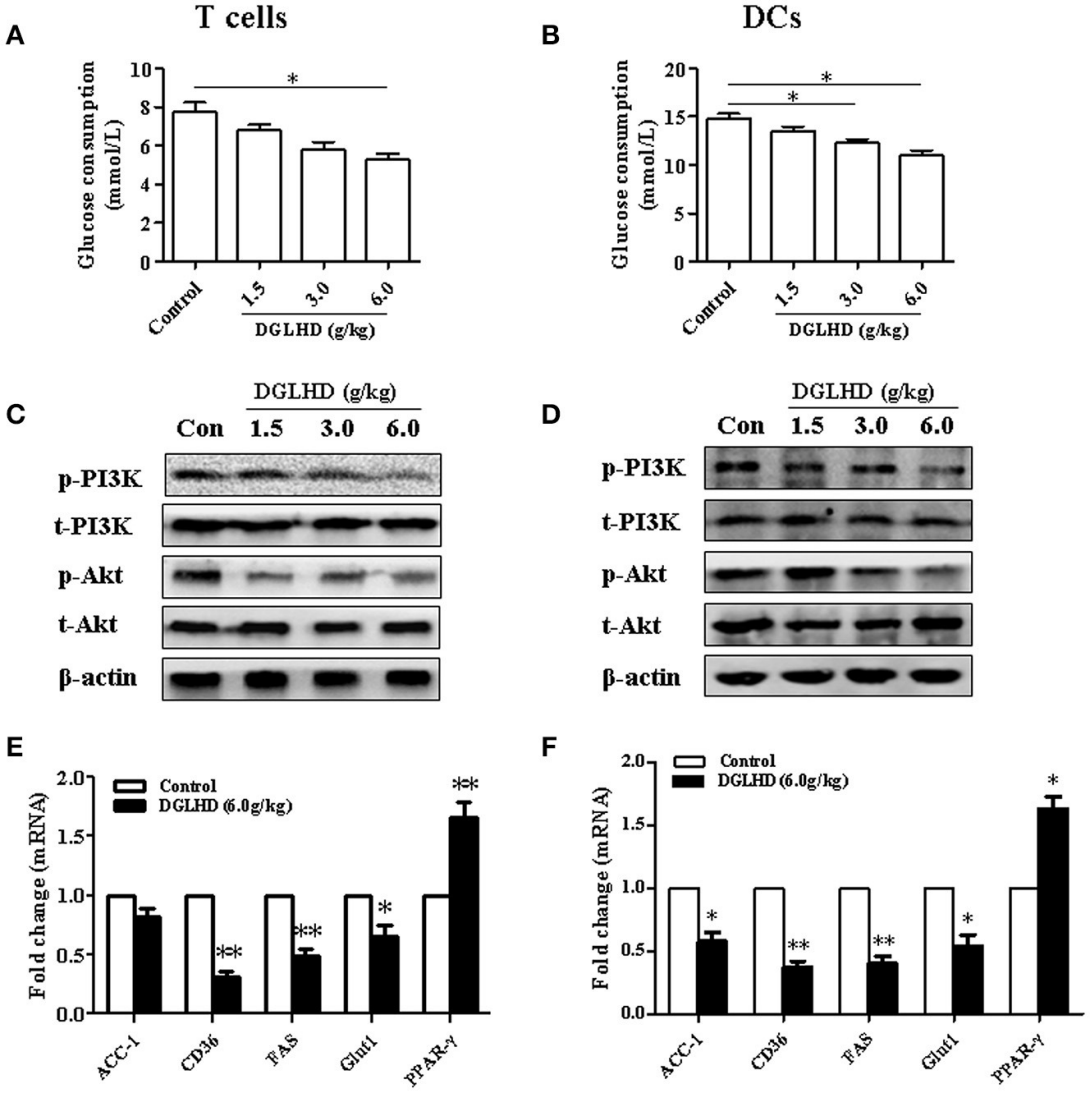
DGLHD alters the metabolic condition of T cells and DCs *in vivo*. T cells and DCs were generated from spleen or bone marrow cells of ob/ob mice given DGLHD or water for 8 weeks. Protein and RNA were isolated from splenic T cells or DCs collected at the indicated times. **(A,B)** Glucose utilization in T cells and DCs. **(C,D)** Protein expressions of PI3K, p-PI3K, Akt, and p-Akt in T cells and DCs. **(E,F)** qRT-PCR analyses of metabolic genes. Data are shown as mean ± SEM (*n* = 4). ^*^P < 0.05, ^**^P < 0.01 vs. Control.

It is becoming clear that changes in cell activation and function coincide with alterations in the cellular metabolism (Pearce and Everts, 2015). Our results documented that DGLHD diminished glucose uptake and the activation of PI3K/Akt signaling pathways in DCs and T cells (Figure 8, Figures S6E–G, S7E–G), then contributing to the enhancement of tolerogenic DCs and Tregs *in vivo* and *in vitro*. Intriguingly, we here found that DGLHD elevated the expression levels of PPAR-γ, accompanied with inhibited lipid biosynthesis in DCs and T cells (Figures 8E,F, Figures S6G, S7G). DGLHD also correspondingly affected the metabolic characteristics of T cells and DCs in Tregs-neutralized ob/ob mice (Figures S5C,D). And DGLHD had a tendency to raise PPAR-γ gene level in PPAR-γ-blocked T cells and DCs (Figures S6H, S7H). These data provided presumptive evidence that DGLHD influenced the function of DCs and T cells through regulating glucolipid metabolism with PI3K/Akt signaling pathway suppression or PPAR-γ activation.

### DGLHD modulates metabolism-related molecules expression of adipocytes and hepatocytes

**Figure 9:**
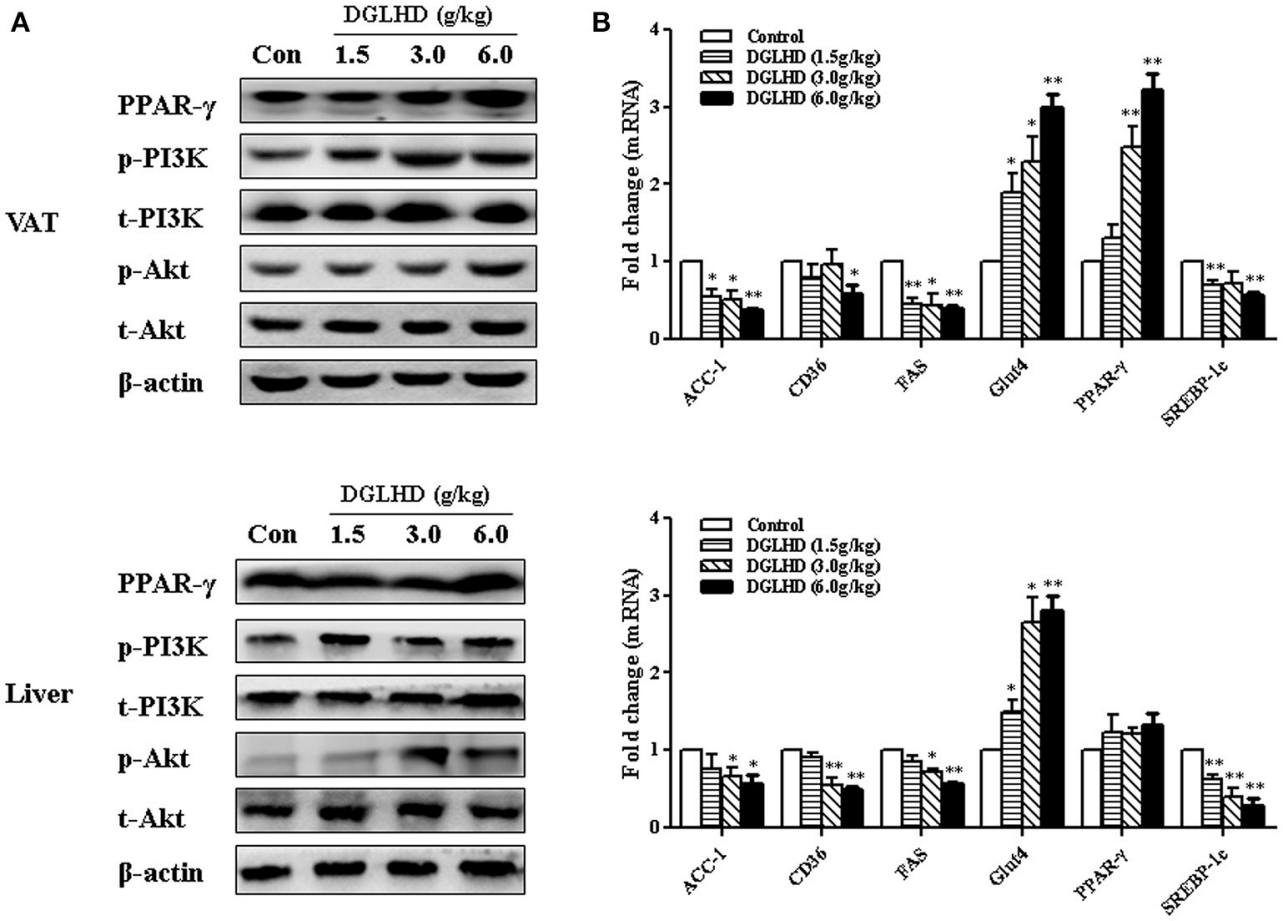
DGLHD regulates metabolic homeostasis via activating PI3K/Akt pathways in visceral adipose and hepatic tissues. Liver and visceral adipose tissues were removed from ob/ob mice given water or DGLHD for 8 weeks. **(A)** Relative protein expressions were analyzed by Western blotting. **(B)** Gene expressions were determined by qRT-PCR analyses. β-actin was detected as the internal reference. Data are shown as mean ± SEM (*n* = 4–6 per group). ^*^P < 0.05, ^**^P < 0.01 vs. Control.

It has been previously reported that the expression of genes associated with lipid biosynthesis—acetyl-CoA carboxylase-1(ACC-1), fatty acid synthase (FAS), and cluster of differentiation 36 (CD36), and sterol regulatory element-binding protein-1 (SREBP-1)—is elevated in adipose and hepatic tissues of obese mice (Hardy et al., 2016). Gene expression analysis demonstrated that DGLHD inhibited these genes expression (Figure 9B). Interestingly, we found that PPAR-γ activity was enhanced by DGLHD in VAT, whereas PPAR-γ level in liver were not significantly affected (Figure 9). While our results also revealed that the PI3K/Akt pathway was activated in both liver and VAT of DGLHD-treated ob/ob mice with improved metabolic state (Figure 9A).

Considering that adipose and liver are major determinants of lipid and glucose metabolism *in vivo*, we examined the direct effects of DGLHD on insulin resistance and steatosis of adipocytes and hepatocytes *in vitro*. In insulin-resistant 3T3-L1 adipocytes, we observed that DGLHD increased glucose uptake, adiponectin levels, PI3K/Akt activation, and attenuated lipid biosynthesis (Figure S8). DGLHD was also inclined to elevate PPAR-γ level in 3T3-L1 adipocytes incubated with PPAR-γ antagonist (Figure S8E). In HepG2 cells, a hepatocyte model of fatty degeneration, DGLHD enhanced glucose utilization and PI3K/Akt signaling, and improved lipogenic genes expression (Figure S9). All of these results are in line with increased insulin sensitivity and improved glucose and lipid metabolism in response to DGLHD.

## Discussion

Obesity and its metabolic abnormalities disrupt immune homeostasis, increase inflammatory cell infiltration, and reduce insulin sensitivity, leading to insulin resistance and liver steatosis; at the same time, persistent inflammation with excessive insulin secretion often results in β cell destruction (Lee and Lee, 2014). However, current pharmacotherapy of type 2 diabetes is not ideal, largely because it does not adequately stem the progression of inflammatory obesity-linked insulin resistance and hepatic steatosis. In the study, we focused on the TCM formula—DGLHD, and possible mechanisms for its ameliorative effects on insulin resistance and hepatic steatosis in ob/ob mice.

The main chemical components in DGLHD detected by HPLC are baicalin, berberine, wogonoside, and wogonin. In TCM, baicalin is employed to treat inflammation, hypertension, cardiovascular diseases, and bacterial and viral infections. Recent studies also indicate that baicalin improves glucose tolerance and insulin resistance, enhances insulin sensitivity, and attenuates lipid deposition, the mechanisms of which are related to the activation of Akt/GSK-3β, Akt/Glut4, and PGC1α/Glut4 pathway as well as enhanced PPAR-γ and p-Akt expression (Xi et al., 2016; Fang et al., 2017). Baicalin also impairs Th1 polarization and prevents the formation of atherosclerotic lesions through decreasing DCs number and inhibiting DCs maturation (Kim et al., 2013; Liu et al., 2014). It has been reported that berberine has many beneficial biological effects, such as anti-inflammatory, anti-insulin resistant, anti-diabetic, hypoglycemic, and hypolipidemic effects. The mechanisms of berberine in diabetes and inflammation include: regulating JNK and PPAR-α pathway, influencing transcriptional factors expression, such as PPAR-γ, C/EBP-α, SREBP-1c, LXR, and modulating IRS-1, Akt, and AMPK signal pathway (Zhang et al., 2008; Lou et al., 2011; Zhao et al., 2012; Deng and Xie, 2014). Additionally, berberine attenuates myocardial ischemia reperfusion injury by tuning the activation of PI3K/Akt signaling (Qin and Yong, 2016). Wogonin has been proved to mitigate inflammation, hyperglycemia, and dyslipidemia, exerting beneficial effects on diabetes and diabetes-associated diseases with elevated adiponectin expression, decreased IL-1β, IL-6, and TNF-α production, and inhibited Th17 differentiation and NF-κB signaling (Bak et al., 2014; Takagi et al., 2014; Wang W. et al., 2015; Khan et al., 2016). Additionally, He et al indicated that Wogonoside possesses anti-oxidant, anti-diabetic, and neuroprotective effects (He et al., 2014). In concert with these findings, we found in this study that DGLHD not only substantially corrected glucose and lipid metabolic abnormalities, but also exhibited anti-inflammatory and immunosuppressive actions, with no obvious adverse effects in DGLHD-treated healthy C57BL/6J mice (Tables S3, S4).

Here, treatment with DGLHD decreased the extent of fat accumulation and improved systemic and local metabolism, as evidenced by lowered fat content in liver and reduced levels of TC, TG, FFA, and increased adiponectin *in vivo* and *in vitro*, the latter of which benefits for improved insulin resistance and steatosis (Tilg and Moschen, 2006; Hardy et al., 2016). Although DGLHD did not markedly alter body weights and food intake of ob/ob mice, it did lead to diminished liver and VAT weights, which was consistent with the finding that DGLHD modulated the expressions of genes involved in lipid biosynthesis in liver and VAT, such as ACC-1, CD36, FAS, SREBP-1c. PPAR-γ is highly expressed in adipose tissue and plays a vital role in glucose and lipid metabolism through harmonizing the actions of multiple players, such as SREBP, PRDM, PGC-1, and several miRNAs (Kim et al., 2015). PPAR-γ upregulation enhances insulin sensitization and correspondingly reduces blood glucose level (Semple et al., 2006). Whereas, hepatocyte specific disruption of PPAR-γ improves fatty liver with reduced insulin sensitivity in diabetic mice (Matsusue et al., 2003). We observed here that DGLHD elevated the expression of PPAR-γ in VAT without affecting that of PPAR-γ in liver. Hence, the ameliorated metabolic state and insulin sensitization initiated by DGLHD may be attributed to PPAR-γ activation in VAT in this model.

Consistent with the genes related with lipid biosynthesis regulated by DGLHD, the metabolites in liver and VAT were also impacted. Specific alterations in metabolite levels may be markers of the progression of diseases involving altered insulin sensitivity. For example, changes in glycine and glutamine levels in plasma correlated negatively with insulin resistance and an increased glutamine/glutamate ratio in plasm predicted a reduced risk of diabetes (Cheng et al., 2012). Elevated concentrations of cholesterol, isoleucine, and tryptophan were identified as strong metabolic predictors of lower insulin sensitivity (Roberts et al., 2014). Interestingly, we found that concentrations of cholesterol, isoleucine, and tryptophan were substantially diminished in liver and VAT of DGLHD-treated ob/ob mice, while glutamine levels were elevated. Pathway-enrichment analysis of these different metabolites showed that DGLHD mainly impacted amino acid and purine metabolism. Specific amino acids are necessary for purine and pyrimidine synthesis, subsequent mRNA production, and regulation of glucose and lipid metabolism (Bruhat et al., 2009). Therefore, improved expression of glucose-metabolic and lipogeneic genes in VAT and liver of DGLHD-treated mice may be causally related to the altered amino acid metabolites in these tissues.

Inflammation and immune response are closely linked to altered metabolic homeostasis in obesity-induced insulin resistance and liver steatosis (Ye, 2013; Lee and Lee, 2014). Our results showed that DGLHD treatment not only suppressed systemic and local inflammation of ob/ob mice, as supported by reduced proinflammatory cytokine levels and increased anti-inflammatory cytokine expression, but also elevated Treg differentiation and impeded DCs maturation and activity in model mice.

Immature DCs typically express low levels of MHC-II and costimulatory molecules to promote T cell anergy and Tregs generation, both of which are important for the maintenance of immune homeostasis (Hubert et al., 2007). On the other hand, mature DCs express high levels of CD86, MHC-II, and IL-12, and stimulate T cell immunity and T cell-proliferative responses (Hubert et al., 2007). In this study, DGLHD treatment of DCs increased PD-L1, IDO, and ILT3 levels, decreased the expressions of CD86 and MHC-II and the secretion of IL-12p70, and decreased DC-mediated T cell proliferation, all characteristics of immature DCs. Therefore, it seems likely that DGLHD attenuates inflammation and immune responses at least partly by retarding the maturation and function of DCs.

Tregs are characterized by their ability to suppress proliferation of effector T cells and induce T cell anergy, which may be beneficial for the improvement of insulin resistance and hepatic steatosis (Ilan et al., 2010). Here, we observed that DGLHD increased Tregs numbers and Foxp3 expression in spleen, lymph node, liver, and VAT from ob/ob mice. Previous reports have suggested that Tregs may sustain the expression of CD86 and CD80 costimulatory molecules on DCs by downregulating the activation of NF-κB, and suppressing effector T cells response by reducing IL-12 production from DCs (Hubert et al., 2007; Larmonier et al., 2007). The suppressive activity of Tregs is mediated by the release of TGF-β and IL-10, which negatively modulates DC maturation and favors the differentiation of tolerogenic DCs, promoting Treg proliferation and responses (Hubert et al., 2007). In the feedback regulatory loop, IDO produced by DCs inhibits T cell proliferation and induces the generation of Tregs. In turn, Tregs can help DCs to express functionally active IDO (Hubert et al., 2007). Additionally, DGLHD also decreased TCR levels and increased PD-1, TGF-β1 and IL-10 expressions in T cells. Activation of T cells requires T cell receptors (TCR) and costimulatory molecules, necessary for communication with antigen presenting cells including DCs (Shepard and Bonney, 2013). Overall, DGLHD may promote not only Treg generation and T cell inefficiency, but also immunosuppress interactions between DCs and Tregs.

Immune cell activation including DCs and Tregs, is coupled to profound changes in metabolism of them, and these metabolic alterations modulate their fate and function (Everts and Pearce, 2014; Pearce and Everts, 2015). PI3K/Akt-dependent signals are key regulators of cellular activation and proliferation largely through control glycolysis and anabolic metabolism (Biswas, 2015). Our results indicated that the protein expression levels of p-PI3K and p-Akt, and glucose consumption, were all suppressed in DCs and T cells treated with DGLHD. The PI3K/Akt pathway also plays a vital role in insulin signal transduction, which is responsible for the integration of lipid and glucose metabolic actions in response to insulin (Lee and Lee, 2014; Zeng et al., 2014). Here, we observed that the activity of the PI3K/Akt pathway was restored in VAT and liver of DGLHD-treated ob/ob mice. Hence, DGLHD treatment optimized differentiation and function of DCs and T cells and affected beneficial metabolic changes in visceral adipose and hepatic tissues probably via regulating PI3K/Akt signaling pathways.

As mentioned above, the enhancement of immature DCs and Tregs initiated by DGLHD is closely associated with the activation of PPAR-γ. PPAR-γ is a key transcription factor controlling metabolism, which negatively affects the maturation of DCs (Kim et al., 2015). Activation of PPAR-γ in DCs decrease cytokine IL-12, chemokine receptor CCR7, and costimulatory molecules CD80 and CD86, and increase the coinhibitory molecule PD-L1 with diminished T cell-priming capacity via promoting fatty acid oxidation and the biological synthesis of mitochondria (Kim et al., 2015; Pearce and Everts, 2015). PPAR-γ also tunes the balance of Th1/Th2 by increasing Th2 cytokine production and decreasing Th1 cytokine production, and promotes the survival and differentiation of regulatory T cells (Kim et al., 2015). Overall, it seems reasonable to speculate that stimulation of PPAR-γ contributes to DGLHD-ameliorated lipid metabolism, promoting DCs and Treg activation and differentiation.

It has been reported that oral DGLHD 15.6 g/day/per subject significant attenuated dental ulcers and gastrointestinal discomfort in patients (Zheng and Quan, 2014). According to the conversion ratio of body surface area from humans to mice, the concentration (3.0 g/kg) used in our study for mice was approximately equivalent to 15 g/day/per subject, which consistent with the previous report (Zheng and Quan, 2014). Liu et al have demonstrated that DGLHD exhibited hypoglycemic and anti-diabetic actions in NOD mice at the doses from 1.25 to 5 g/kg (Liu et al., 2015). In conclusion, our research about DGLHD and its concentrations selection in ob/ob mouse model will provide a reference for patients who use DGLHD to treat obesity-related insulin resistance and hepatic steatosis in the future.

In summary, DGLHD exerts beneficial effects on immune and metabolic balance through regulating PI3K/Akt pathway or PPAR-γ, playing potent anti-inflammatory, anti-insulin resistant, and anti-steatotic effects in ob/ob mice. DGLHD directly suppressed the activation and function of DCs, and promoted Treg generation and DCs-Treg interactions in ob/ob mice with insulin resistance and steatosis by altering their metabolism. DGLHD also favorably modulated metabolites level associated with the development of insulin resistance in VAT and liver. These results provide experimental evidence for the therapeutic utility of DGLHD as an agent against insulin resistance and hepatic steatosis in humans. Clearly identifying the active components in the DGLHD concoction is a subject of future investigations.

## Prior presentation

Parts of this study has been submitted in abstract form at IMMUNOLOGY 2017™, Washington, D.C., May 12-16, 2017.

## Author contributions

HC drafted the manuscript and prepared the figures. HC and LT performed experiments, analyzed and interpreted the data. YT, ZX, and RF performed experiments. YL, YQ, JL, and ZY provided suggestions and material support. MX designed the study, obtained the funding, supervised the whole project and reviewed the manuscript.

### Conflict of interest statement

The authors declare that the research was conducted in the absence of any commercial or financial relationships that could be construed as a potential conflict of interest.
