# Supplementary material for: Immune and Metabolic Regulation Mechanism of Dangguiliuhuang Decoction against Insulin Resistance and Hepatic Steatosis
Source: Front Pharmacol. 2017 Jul 7;8:445. doi: 10.3389/fphar.2017.00445 (PMC5500616; doi:10.3389/fphar.2017.00445)
Supplement: Supplementary file 1 [file Presentation1.pdf]

Supporting Information

Immune and Metabolic Regulation Mechanism of Dangguiliuhuang Decoction Against Insulin Resistance and Hepatic Steatosis

Hui Cao[1#], Lingling Tuo[2#], Yali Tuo[1], Ziyun Xia[3], Rong Fu[1], Yang Liu[4], Yihong Quan[2], Jue Liu[2], Zhihong Yu[2], Ming Xiang[1]*

[1]Department of Pharmacology, School of Pharmacy, Tongji Medical College, Huazhong University of Science and Technology, Wuhan, China

[2]Department of Traditional Chinese Medicine, the Central Hospital of Wuhan, Tongji Medical College, Huazhong University of Science and Technology, Wuhan, China

[3]Department of Pharmacy, China Pharmaceutical University, Nanjing, China

[4]Synergy Innovation Center of Biological Peptide Antidiabetics of Hubei Province, School of Life Science, Wuchang University of Technology, Wuhan, China

***Correspondence:**

Ming Xiang

E-mail: xiangming@mails.tjmu.edu.cn

[#]These authors contributed equally to this work

Table S1 | Herbal ingredients in the traditional Chinese medicine formula-DGLHD

Scientific Name	Chinese Name	Traditional Use or Pharmacology
Angelica sinensis	Dang Gui	Used to treat inflammation, cardiovascular conditions, osteoarthrosis, headache, infections, mild anemia, fatigue and high blood pressure
Phellodendron amurense	Huang Bai	Used to treat meningitis, bacillary dysentery, pneumonia, tuberculosis, and liver cirrhosis; Used orally to treat abdominal pain, diarrhoea, gastroenteritis and urinary tract infections; prevent osteoarthritis, lung or prostate tumor progression; help overweight/obese people.
Scutellaria baicalensis	Huang Qin	Used to inhibit inflammation, hepatitis, and diarrhea; treat gynaecological conditions; treat anxiety and muscle tension.
Coptis chinensis	Huang Lian	Used to against intestinal injury, atherosclerosis, neurodegeneration, the pain of irritable bowel syndrome
Astragalus membranaceus	Huang Qi	Used to treat diabetes, heal and seasonal allergic rhinitis
Radix Rehmanniae	Sheng Di Huang	Used to treat exogenous heat
Rehmannia glutinosa	Shu Di Huang	Used to treat diabetes mellitus, tinnitus deafness, dizzy head and vision, premature graying in beard and hair, constipation, kidney vacuity hasty asthma.

Table S2 | Forward and reverse primers applied to quantitative real-time PCR analysis

**Gene**	**Forward primer**	**Reverse primer**
ACC-1	GAGTGACTGCCGAAACATCTCTG	GCAAGGAGGACAGAGTTTATCGTG
Adiponectin	GCACTGGCAAGTTCTACTGCAA	GTAGGTGAAGAGAACGGCCTTGT
CD36	TTGGCCAAGCTATTGCGACA	GCAAAGGCATTGGCTGGAAG
FAS	GCTGCGGAAACTTCAGGAAAT	AGAGACGTGTCACTCCTGGACTT
Foxp3	ATCTCCTGGATGAGAAAGGCAAGG	AGAGCTCTTGTCCATTGAGGCCA
Glut1	CAGTTCGGCTATAACACTGGTG	GCCCCCGACAGAGAAGATG
Glut2	ATCCCTTGGTTCATGGTTGCTG	TCCGCAATGTACTGGAAGCAG
Glut4	GTGACTGGAACACTGGTCCTA	CCAGCCACGTTGCATTGTAG
IDO	TTGCCAAATGTGACTGCT	CAAACTAATGACAAATCCCT
IFN-γ	CTTTGCAGCTCTTCCTCATGGCTGTTTCTG	TGACGCTTATGTTGTTGCTGATGGCCTG
IL-1β	CCAGCTTCAAATCTCACAGCAG	CTTCTTTGGGTATTGCTTGGGATC
IL-6	ACAACGATGATGCACTTGCAGA	GATGAATTGGATGGTCTTGGTC
IL-10	TGGCCCAGAAATCAAGGAGC	CAGCAGACTCAATACACACT
ILT-3	AAAGGCTGGAACTATGAG	AGGAGACAATGAGGTGGA
PPAR-γ	GTGCCAGTTTCGATCCGTAGA	GGCCAGCATCGTGTAGATGA
SREBP-1c	GATGTGCGAACTGGACACAG	CATAGGGGGCGTCAAACAG
TGF-β1	CACTGATACGCCTGAGTG	GTGAGCGCTGAATCGAAA
TNF-α	CACAGAAAGCATGATCCGCGACGT	CGGCAGAGAGGAGGTTGACTTTCT
hTNF-α	CCAGGCAGT CAGATCATCTTCTC	AGCTGGTTATCTCTCAGCTCCAC
hIL-6	CAATCTGGATTCAATGAGGAGAC	TCTGGCTTGTTCCTCACTACTC
β-actin	TGTGATGGTGGGAATGGGTCAG	TTTGATGTCACGCACGATTTCC
hβ-actin	TCAGAAGGATTCCTATGTGGGCGA	TTTCTCCATGTCGTCCCAGTTGGT

Table S3 | Serum biochemistry levels in C57BL/6J mice treated with water or DGLHD

Group	Body weight (g)	Liver weight (g)	TG (mM)	TC (mM)	FFA(μM)	ALT(U/L)	AST(U/L)
**Control**	23.67±1.36	1.07±0.11	1.57±0.36	1.48±0.23	120.78±23.87	29.49±4.37	82.93±12.87
**Control+DGLHD**	23.40±1.83	1.11±0.08	1.51±0.28	1.41±0.45	130.56±21.29	30.56±5.24	90.16±10.36

Data are expressed as mean ± SEM (n=5). TG: triglyceride; TC: total cholesterol; FFA: free fatty acid; ALT: alanine aminotransferase; AST: aspertate aminotransferase.

Table S4 | Effect of DGLHD on serum cytokines level in C57BL/6J mice treated with water or DGLHD

Group	IL-6 (pg/ml)	IL-10 (pg/ml)	IFN-γ (pg/ml)	TGF-β_1 (pg/ml)	TNF-α (pg/ml)	Insulin (ng/ml)
**Control**	198.13±57.86	75.56±5.94	304.43±39.58	1662.61±180.76	87.11±21.45	0.53±0.11
**Control+DGLHD**	216.58±51.92	73.72±9.31	314.94±33.02	1707.63±159.85	88.85±20.24	0.50±0.09

Data are expressed as mean ± SEM (n=5). IL-6: interleukin-6; IL-10: interleukin-10; IFN-γ: interferon—γ; TGF-β1: transforming growth factor-β1; TNF-α: tumor necrosis factor-α.

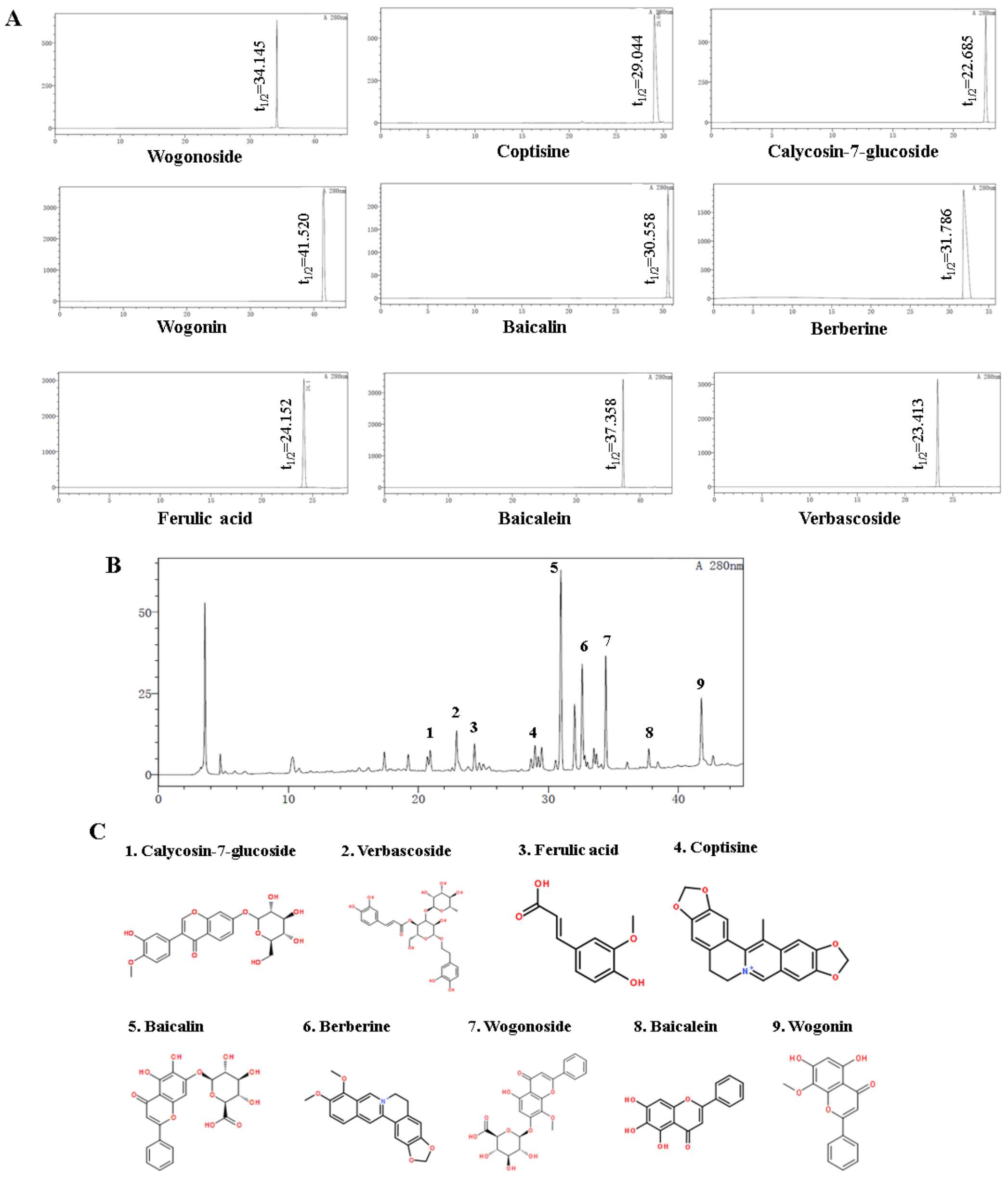

Figure S1 | HPLC chromatogram and chemical structure of the main compounds in DGLHD. (A) Representative chromatograms of primary compounds in DGLHD. (B) Representative HPLC chromatogram of DGLHD. (C) Chemical structure of principal active components.

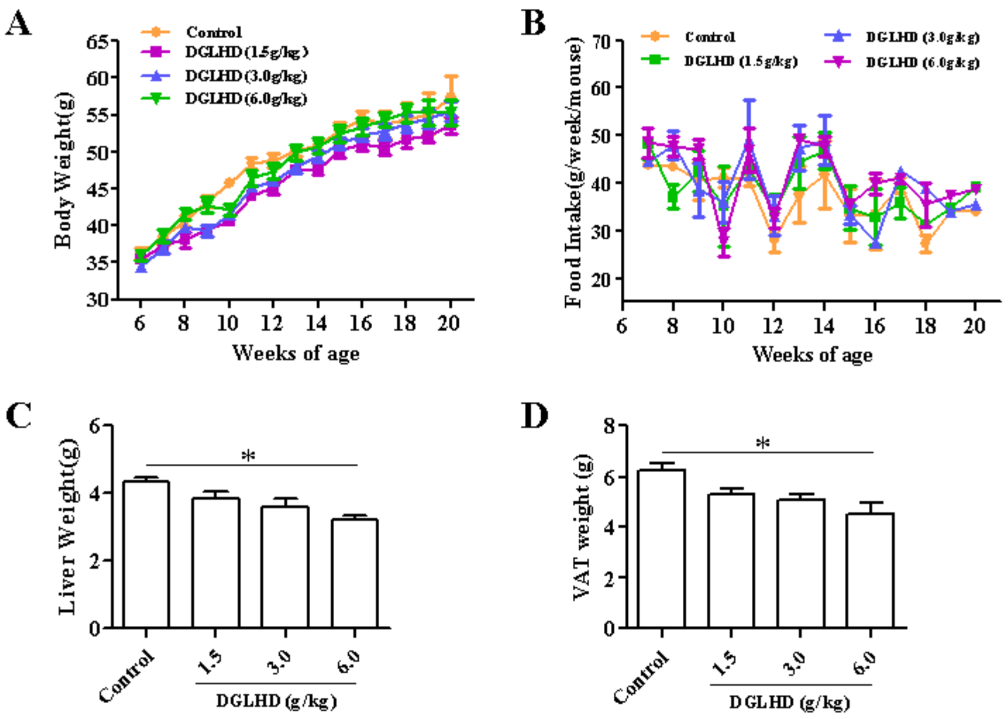

Figure S2 | DGLHD decreased the weight of visceral adipose tissue (VAT) and liver in ob/ob mice. Ob/ob mice were treated daily with water or DGLHD at 1.5, 3.0 or 6.0g/kg by intragastric gavage for 8 weeks (n=5 for each group). Body weight (A) and food intake (B) were assessed in mice. The weights of liver (C) and VAT (D) were shown. Data are represented as mean±SEM. Statistical significance was determined by Student's t test. **P* < 0.05 vs. Control.

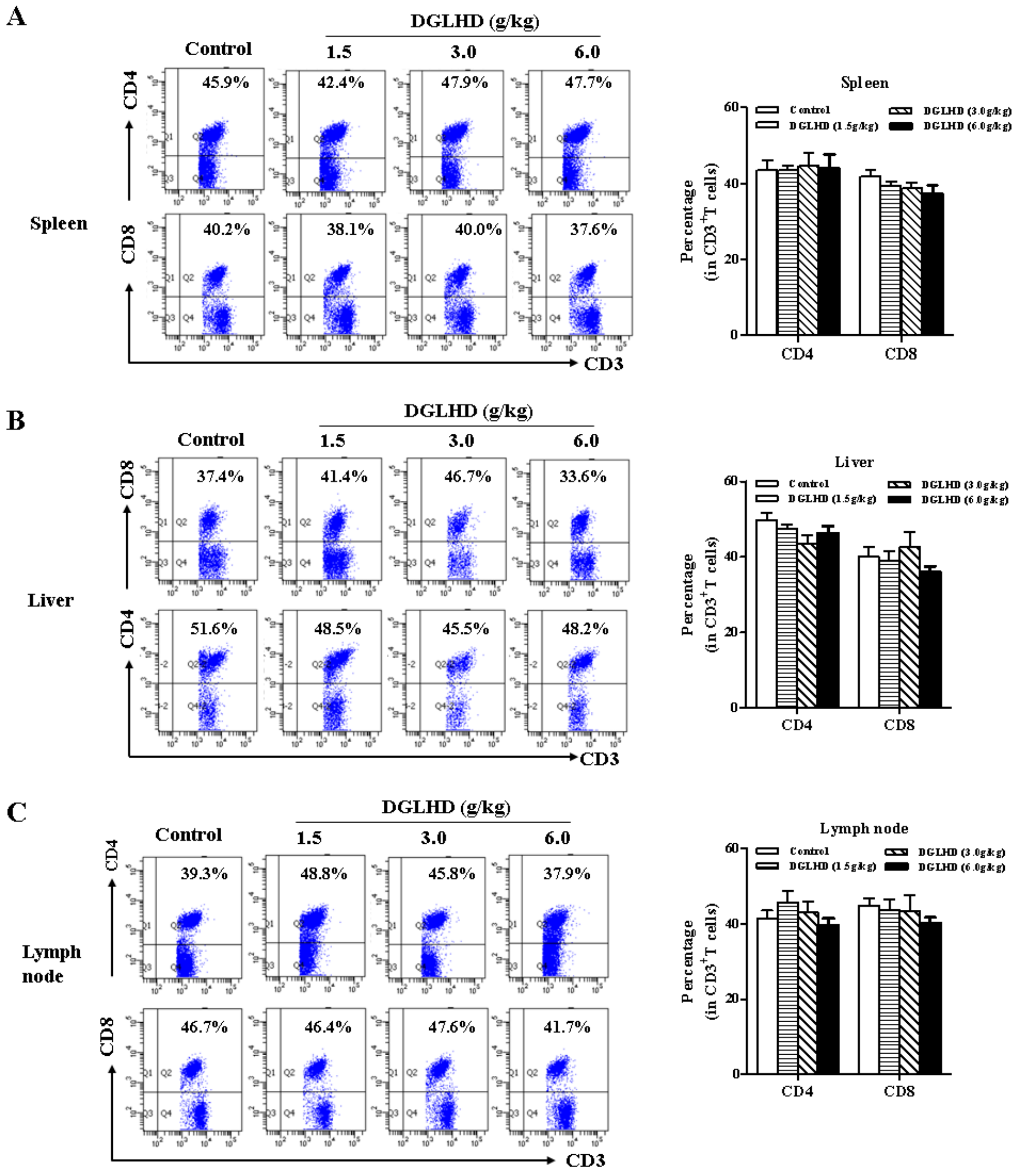

Figure S3 | DGLHD has no apparent effects on the proportion of CD4$^+$ and CD8$^+$T cells. Spleen, lymph node, and liver were derived from ob/ob mice. (A-C) Representative FACS staining for CD4 and CD8 on gated CD3$^+$T cells and the percentage of CD4$^+$ and CD8$^+$ T cells in spleen (A), liver (B) and lymph node (C). Data are expressed as mean±SEM (n = 3-4).

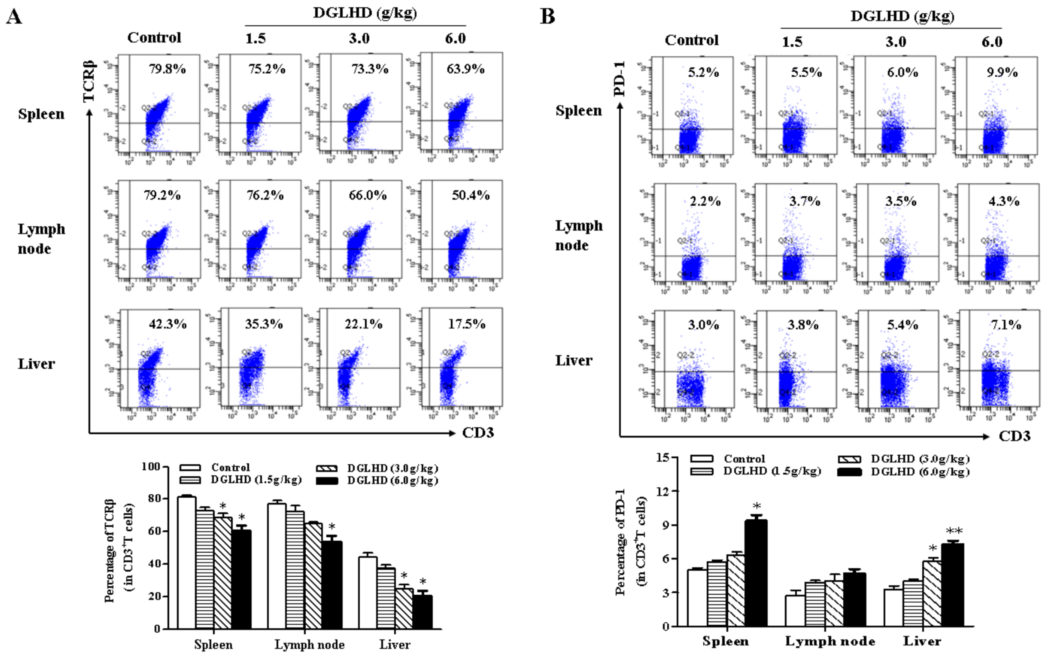

Figure S4 | Effects of DGLHD on TCRβ and PD-1 expression in T cells. Spleen, lymph node and liver were generated from 14 week-old ob/ob mice. Expression levels of TCRβ (A) and PD-1 (B) in $CD3^{+}$T cells from spleen, lymph node and liver were detected by FCM. Data are represented as mean±SEM (n = 4). **P* < 0.05, ***P* < 0.01 vs. Control.

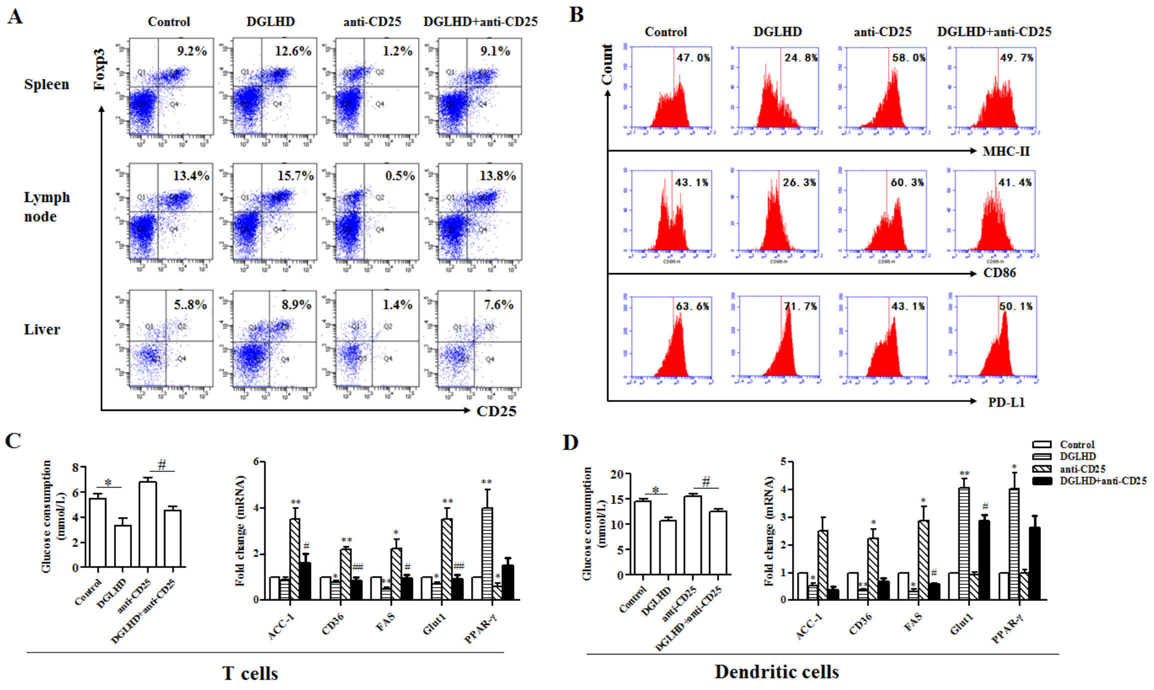

Figure S5 | DGLHD altered the function and metabolism of T cells and DCs in Tregs-depleted ob/ob mice. Tregs in ob/ob mice were neutralized with anti-CD25 antibody. (A) Tregs percentage in $CD4^{+}T$ cells were analyzed by FCM. (B) The levels of CD86, MHC-II and PD-L1 in DCs were examined by FCM. Glucose utilization and relative expression of genes in T cells (C) and DCs (D) from ob/ob mice. Data are expressed as mean±SEM (n = 3-4). $^{*}P < 0.05$, $^{**}P < 0.01$ vs. Control; $^{\#}P < 0.05$, $^{\#\#}P < 0.01$ vs. anti-CD25 group.

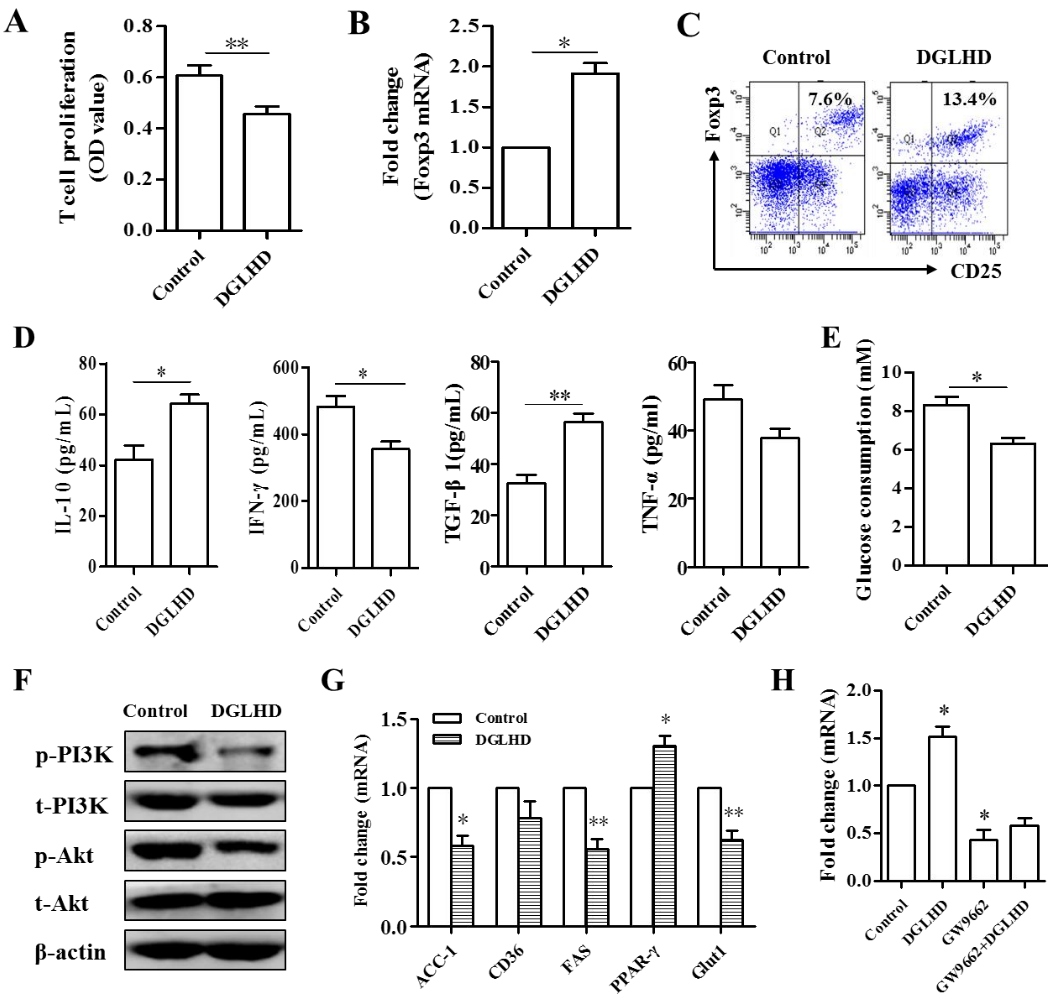

Figure S6 | DGLHD induces Tregs differentiation through altered T cell metabolism *in vitro*. CD4$^+$T cells were isolated from the spleen of ob/ob model mice by anti-CD4 immunomagnetic beads, then incubated with DGLHD (5mg/mL) or/and PPAR-γ antagonist - GW9662 (25μM) for 24 hours. (A) DGLHD suppressed T cell proliferation. (B) Foxp3 mRNA level in T cells. (C) Tregs proportion in CD4$^+$T cells were measured by FCM. (D) The secretion of IL-10, IFN-γ, TGF-β1, and TNF-α in CD4$^+$T cells suspension were assessed by ELISA. (E) Glucose consumption of CD4$^+$T cells. (F) Proteins expression of PI3K, p-PI3K, Akt and p-Akt were examined in CD4$^+$T cells by Western blotting. (G) Metabolic-related genes expression in CD4$^+$T cells. (H) PPAR-γ mRNA level in CD4$^+$T cells. Data are presented as mean±SEM (n=3). *P < 0.05, **P < 0.01 vs. Control.

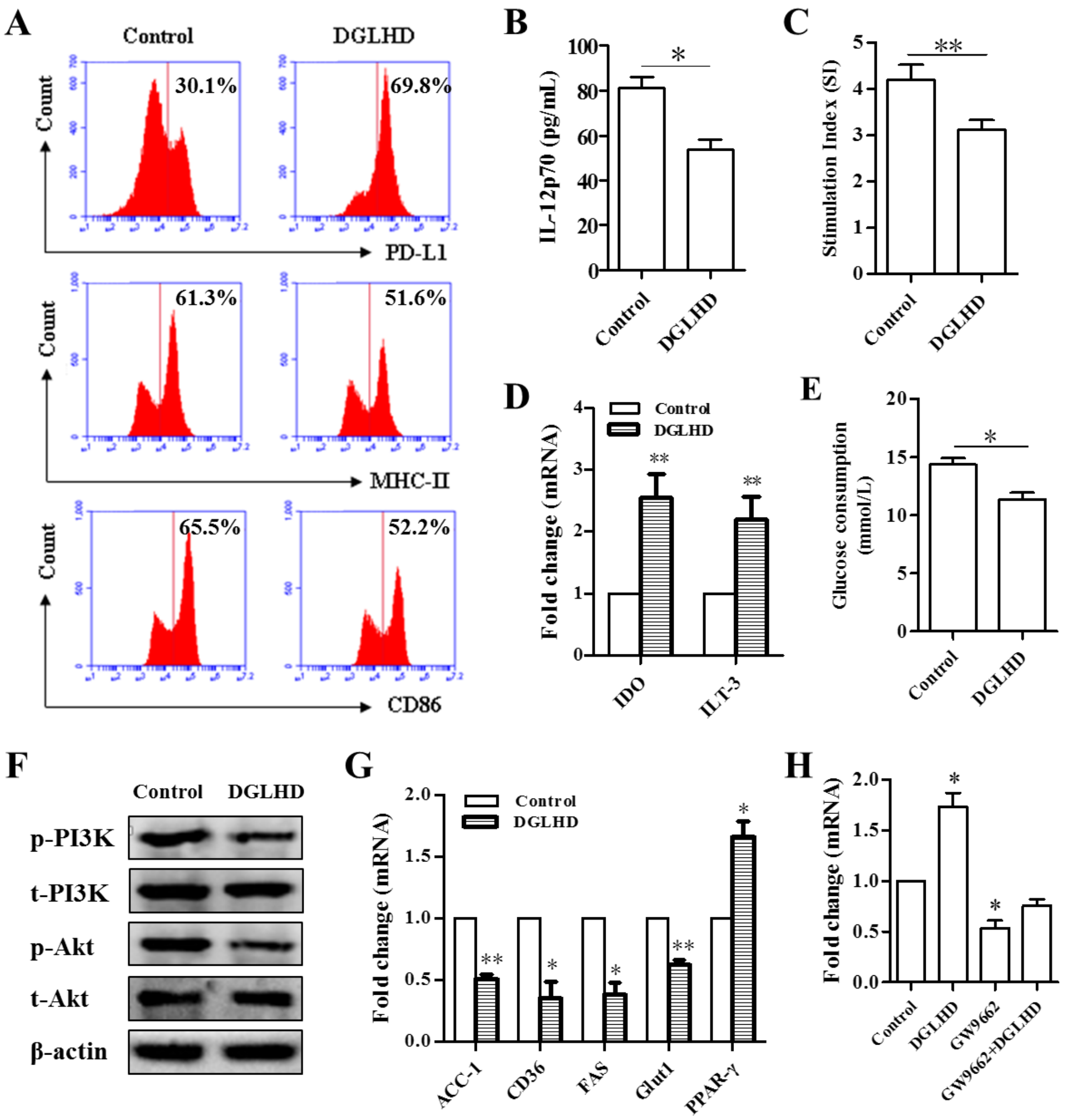

Figure S7 | DGLHD regulates the maturation and function of DCs via changing its metabolic characteristics *in vitro*. Bone marrow dendritic cells were obtained from ob/ob mice, then incubated with DGLHD (5mg/mL) or/and GW9662 (25μM) for 24 hours. (A) Expression of PD-L1, MHC-II, and CD86 in DCs were determined by FCM. (B) Secretion of IL-12p70 cytokine from DCs was assessed by ELISA. (C) The capacity of DCs stimulating T cells proliferation in MLR. (D) Gene levels of IDO and ILT3 were detected by qRT-PCR. (E) Glucose consumption in DCs. (F) Protein levels were measured by Western blotting. (G) Metabolic-related gene expression in DCs. (H) The gene level of PPAR-γ in DCs. Data are shown as mean±SEM (n=3). *$P < 0.05$, **$P < 0.01$ vs. Control.

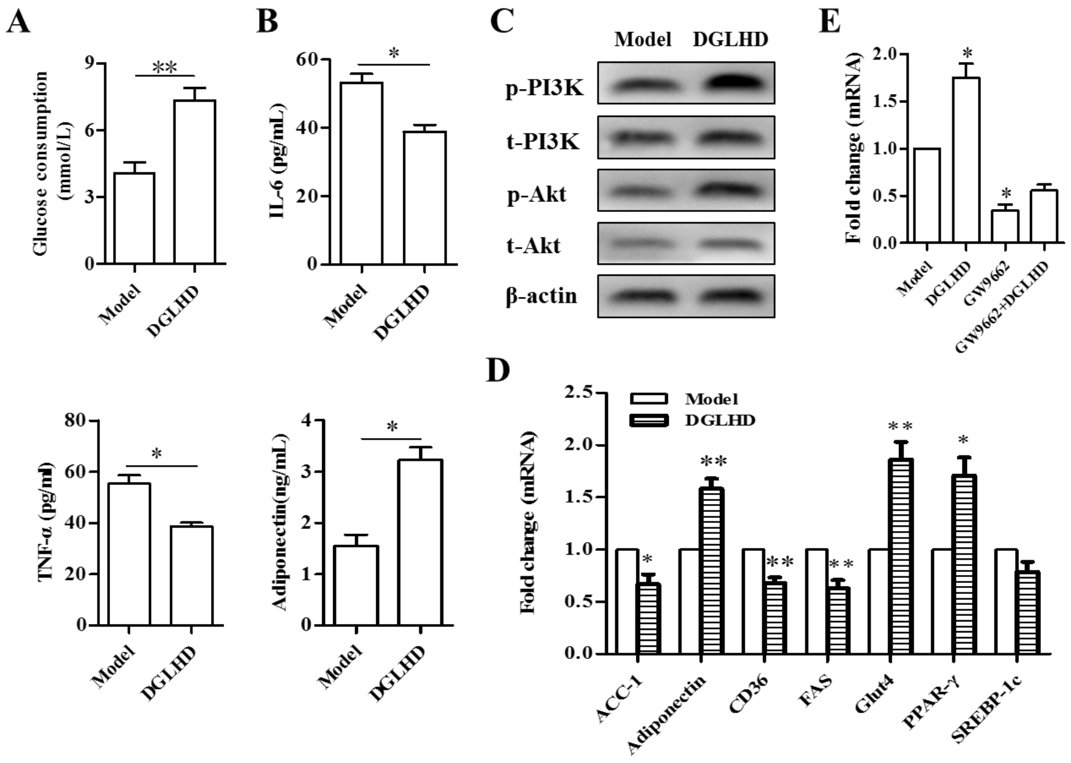

Figure S8 | DGLHD relieves the insulin resistance of 3T3-L1 adipocytes. 3T3-L1 preadipocytes were induced into insulin resistant adipocytes, then co-cultured with DGLHD (5mg/mL) or/and GW9662 (25μM) for 24 hours. (A) Glucose uptake of 3T3-L1 cells. (B) Concentration of IL-6, TNF-α and adiponectin in adipocytes suspension were evaluated by ELISA. (C) Proteins level of PI3K, p-PI3K, Akt and p-Akt were detected by Western blotting. (D) Genes expression level were analyzed by qRT-PCR. (E) PPAR-γ gene expression level. Data are expressed as mean±SEM (n=3). *$P < 0.05$, **$P < 0.01$, compared with Model.

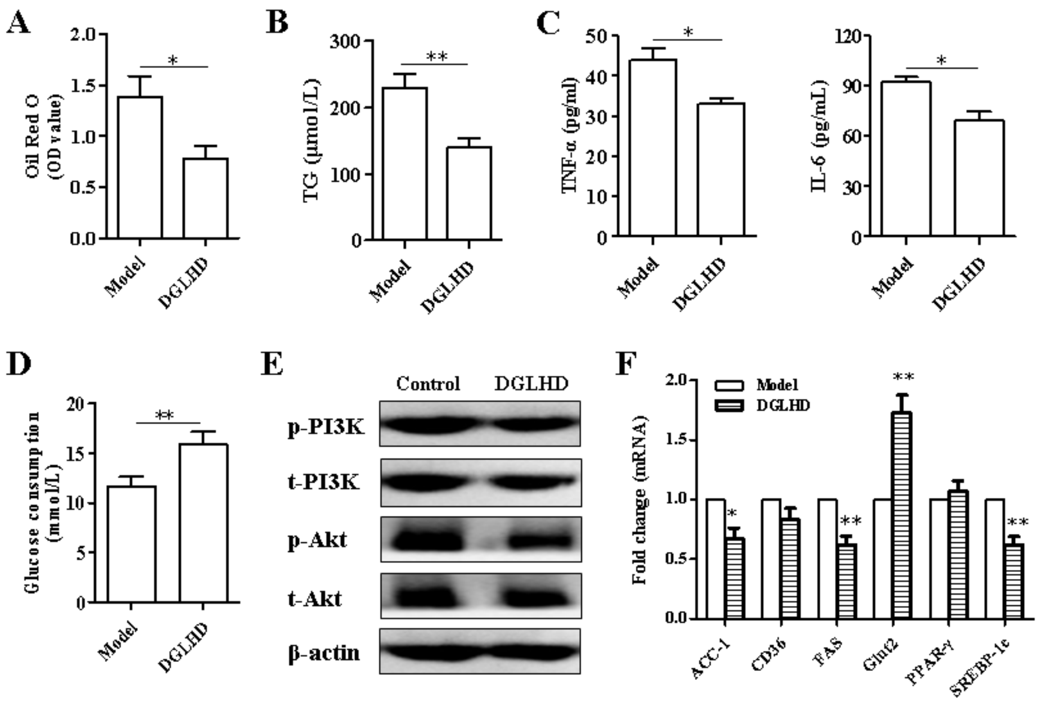

Figure S9 | DGLHD attenuates steatosis in HepG2 cells. HepG2 cells were constructed a steatosis-like status, and incubated with DGLHD (5mg/mL) for 24 hours at the same time. (A) The lipid content in steatotic cells were measured by Oil Red O staining. (B) TG level was quantified by GPO-PAP method. (C) Production of IL-6 and TNF-α cytokines in cells supernatant. (D) Glucose consumption of HepG2 cells. (E) Protein levels were examined by Western blotting. (F) qRT-PCR was used to analyze gene expression. Data are shown as mean±SEM (n=3). *P < 0.05, **P < 0.01, compared with Model.
